# A BPTF-specific PROTAC degrader enhances NK cell-based cancer immunotherapy

**DOI:** 10.1016/j.ymthe.2025.02.013

**Published:** 2025-02-11

**Authors:** Yunjia Li, Lin Bai, Hao Liang, Peidong Yan, Hao Chen, Zhuoxian Cao, Yiqing Shen, Zhongyv Wang, Mei Huang, Bin He, Quan Hao, Yide Mei, Haiming Wei, Chen Ding, Jing Jin, Yi Wang

**Affiliations:** 1 Department of Hepatobiliary Surgery, The First Affiliated Hospital of USTC, Key Laboratory of Immune Response and Immunotherapy, Center for Advanced Interdisciplinary Science and Biomedicine of IHM, School of Basic Medical Sciences, Division of Life Sciences and Medicine, University of Science and Technology of China, Hefei 230027, China; 2 State Key Laboratory of Genetic Engineering, Institutes of Biomedical Sciences, Human Phenome Institute, School of Life Sciences, Zhongshan Hospital, Fudan University, Shanghai 200433, China; 3 State Key Laboratory of Functions and Applications of Medicinal Plants, Engineering Research Center for the Development and Application of Ethnic Medicine and TCM (Ministry of Education), Guizhou Provincial Key Laboratory of Pharmaceutics, School of Pharmacy, Guizhou Medical University, Guiyang 550004, China; 4 School of Biomedical Sciences, University of Hong Kong, Hong Kong, China

**Keywords:** epigenetic modification, PROTACs, natural killer cells, immunotherapy, hepatocellular carcinoma

## Abstract

Natural killer (NK) cell-based immunotherapy shows promise in cancer treatment, but its efficacy remains limited, necessitating the development of novel strategies. In this study, we demonstrate that the epigenetic factor bromodomain PHD-finger containing transcription factor (BPTF) hinders hepatocellular carcinoma (HCC) recognition by NK cells through its PHD finger’s interpretation of H3K4me3. We have generated a small-molecule proteolysis-targeting chimera (PROTAC) that selectively degrades human and murine BPTF. The degradation of BPTF using PROTACs directly enhances the abundance of natural cytotoxicity receptor ligands on HCC cells, facilitating their recognition by NK cells and thereby augmenting NK cell cytotoxicity against HCC both *in vitro* and *in vivo*. Through multidisciplinary techniques, our findings establish targeting BPTF with PROTACs as a promising approach to overcome immune evasion of HCC from NK cells and provide a new strategy to enhance NK cell-based cancer immunotherapy.

## Graphical abstract

**Figure:**
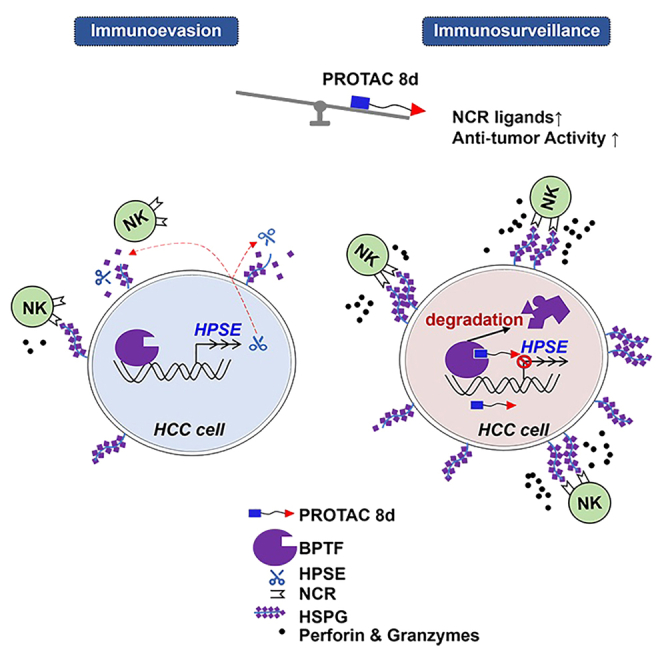

## Teaser

Elevated BPTF enables HCC to evade the immune system. Wang and colleagues have developed PROTAC **8d**, which selectively degrades BPTF, leading to an increase in recognition ligands on HCC cells, thereby enhancing the destruction by NK cells, effectively restoring NK cells' ability to combat cancer.

## Introduction

Hepatocellular carcinoma (HCC) is characterized by its aggressive nature and the limited efficacy of available treatments, particularly in advanced stages.^1^ Immunotherapy has emerged as a promising approach for advanced HCC treatment, supported by both pre-clinical and clinical data.^2^^,^^3^ Among the immune systems involved in combating HCC, natural killer (NK) cells stand out due to their significant presence, accounting for 25%–50% of the liver’s lymphocyte population.^4^ This highlights their crucial role in the development of immunotherapies for HCC.^5^^,^^6^ Moreover, increased quantity and enhanced functionality of NK cells within peripheral blood and tumor tissues are observed in patients with HCC, indicating better survival prospects and a more favorable prognosis.^7^ Therefore, NK cell-based immunotherapy holds substantial potential for treating HCC. The effectiveness of this therapeutic approach relies on activating receptors engagement on NK cells such as natural cytotoxicity receptors (NCRs), which interact with ligands presented on the surface of HCC cells.^8^^,^^9^ This interaction plays an instrumental role in triggering the NK cell-mediated cytotoxicity and cytokine release that contribute to the anti-HCC response.^6^^,^^10^ However, HCC cells may evade NK cell-mediated immunosurveillance by downregulating the expression of ligands crucial for recognition by NK cells.^11^

The abundance of ligands for NK cell-activating receptors on tumor cells can be dynamically modulated through epigenetic mechanisms.^12^^,^^13^ Bromodomain PHD-finger containing transcription factor (BPTF), a core subunit of the ATP-dependent nucleosome remodeling factor complex, functions as an epigenetic reader and is significantly overexpressed in various types of cancer, including liver cancer, lung cancer, and breast cancer, among others.^14^^,^^15^^,^^16^ Its heightened expression plays a crucial role in promoting cancer development by influencing chromatin structure and regulating gene transcription.^12^^,^^14^^,^^17^^,^^18^ Interestingly, knockdown (KD) of BPTF in breast cancer cells leads to a reduction in heparanase (HPSE) expression, thereby compromising HPSE’s ability to cleave heparan sulfate proteoglycans (HSPGs), which serve as NCR ligands on the surface of tumor cells.^12^ The disruption in HSPG cleavage renders the tumor cells more susceptible to elimination by NK cells. This suggests that highly selective inhibitors targeting BPTF could potentially enhance the efficacy of NK cell-based anti-tumor responses.

Recent studies have identified potential inhibitors targeting BPTF bromodomains.^19^^,^^20^^,^^21^^,^^22^ However, as existing compounds may not fully suppress the functions of other domains such as the PHD finger, an alternative strategy focusing on degrading BPTF could more effectively inhibit its overall function. Proteolysis-targeting chimeras (PROTACs) have emerged as a novel and promising strategy that selectively degrade target proteins through the ubiquitin-proteasome pathway.^23^ Comprising an E3 ubiquitin ligase-binding domain, a protein of interest (POI) binding domain, and a linker, PROTACs catalyze the ubiquitination and subsequent proteasomal degradation of the POI.^23^ The advantage of PROTACs lies in their ability to deliver more potent and enduring inhibitory effects compared with conventional small molecule inhibitors.^24^ Furthermore, their design mitigates the risk of off-target interactions, which often contribute to undesired side effects.^23^^,^^25^^,^^26^ Additionally, PROTAC technology functions by inducing the degradation of target proteins rather than merely inhibiting their specific domain functions.^27^ Consequently, using the PROTAC approach has significant potential in facilitating the development of highly selective BPTF inhibitors. Pomerantz and his colleagues^28^ have dedicated considerable effort to this field, resulting in the successful development of PROTACs that targets CECR2 or BPTF for degradation.

Our objective was to elucidate the precise role of BPTF as a histone mark reader in initiating HPSE expression within HCC cells. Subsequently, we aimed to leverage PROTAC technology for designing and synthesizing a series of degraders to efficiently degrade BPTF and effectively diminish its overall function. The efficacy of these PROTACs in enhancing NK cell recognition and eradication of HCC cells both *in vitro* and *in vivo* was then evaluated.

## Results

### BPTF regulates *HPSE* expression and correlates with H3K4me3

**Figure 1:**
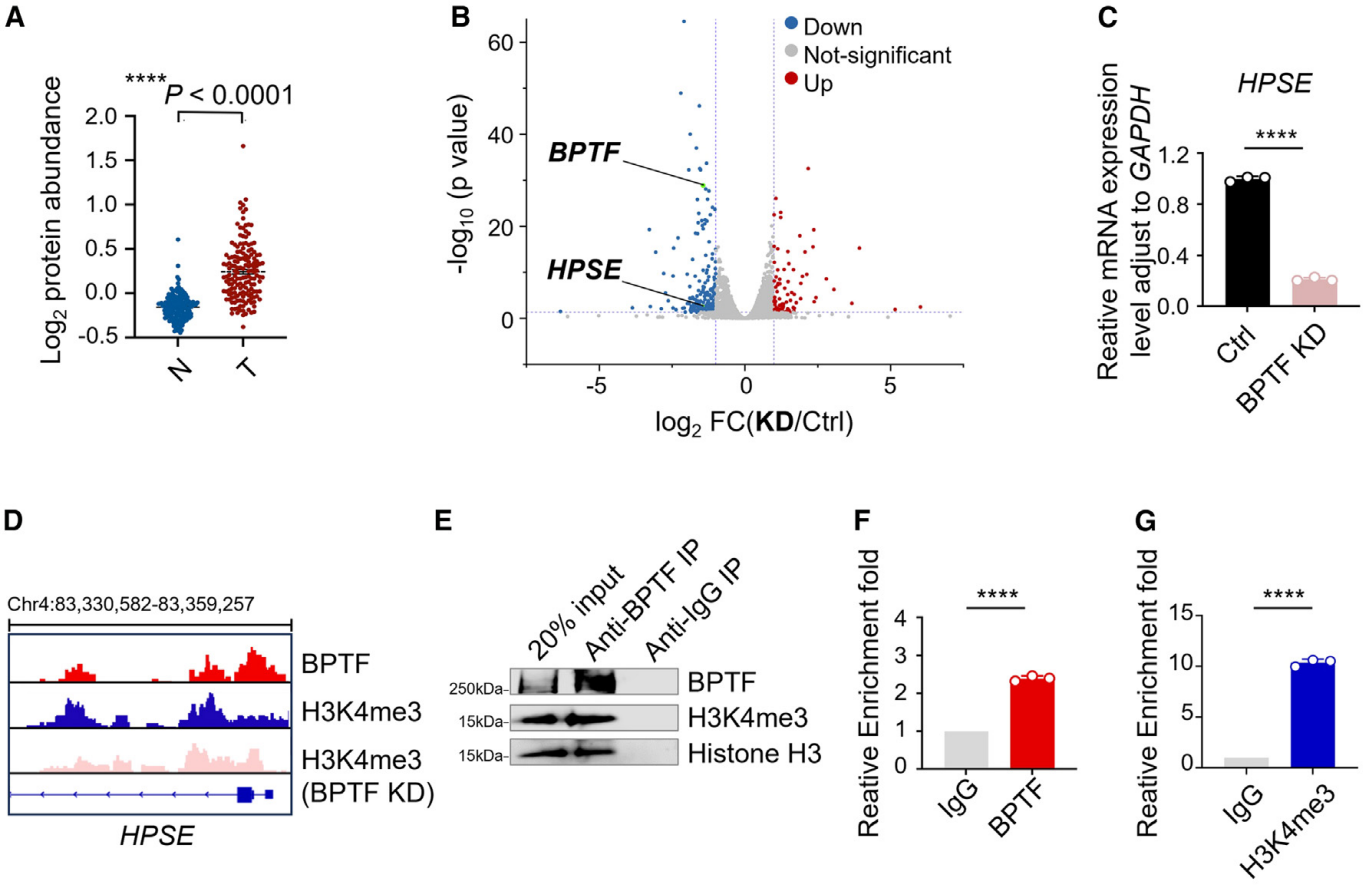
*HPSE* expression is regulated through BPTF’s recognition of H3K4me3 (A) Comparisons of the protein abundance of BPTF between adjacent normal tissue (N) samples and tumor (T) (paired t test) in the HCC patients’ cohort (*n* = 159). The line represents mean with SEM and upper and lower quartiles, respectively. The *p* value is depicted in the figure. (B) Volcano plot illustrating differentially expressed gene sets that are significantly down-regulated or up-regulated in Huh7 cells in response to BPTF silencing according to RNA-seq data; comparison between two biological replicates (*n* = 2). (C) RT-qPCR analysis showing changes in *HPSE* mRNA levels in Huh7 cells with or without KD of BPTF. The RT-qPCR signal was normalized by *GAPDH* (*n* = 3). (D) Genome browser view of CUT&Tag sequencing signal on the chromosome 4 segment, showing the localization of BPTF (depicted in red) and H3K4me3 (shown in blue) binding peaks at the promoter region of *HPSE* in Huh7 cells; and H3K4me3 (shown in pink) binding peaks at the promoter region of *HPSE* in BPTF KD Huh7 cells. (E) IP assay using anti-BPTF antibody and IgG control to confirm the endogenous association between BPTF with H3K4me3 in Huh7 cells. The displayed image is representative of three biological replicates. (F) CUT&Tag qPCR analysis using anti-BPTF antibody and IgG control to detect the enrichment of BPTF at the promoter region of *HPSE* in Huh7 cells (*n* = 3). (G) CUT&Tag qPCR analysis using anti-H3K4me3 antibody and IgG control to detect the enrichment of H3K4me3 at the promoter region of *HPSE* in Huh7 cells (*n* = 3). The data in (C), (F), and (G) were presented as mean ± SD; ∗*p* < 0.05, ∗∗*p* < 0.01, ∗∗∗*p* < 0.001, ∗∗∗∗*p* < 0.0001; unpaired t test.

We initially examined the expression of BPTF in a cohort of 159 HCC patients from Gao et al.'s study.^29^ Our proteomics analysis and immunoblotting results revealed significantly higher BPTF expression in tumor tissues compared with adjacent normal tissues (Figures 1A and S1A). Moreover, BPTF expression were slightly higher in patients with advanced-stage HCC relative to those with early-stage HCC (Figure S1B), indicating a positive correlation between BPTF expression and HCC progression. Furthermore, we carried out survival analysis on 182 HCC patients using the GEPIA2 database, which demonstrated that heightened BPTF expression is associated with inferior disease-free survival in HCC patients (Figure S1C). These findings suggest that excessive BPTF expression may serve as a prognostic indicator for poor outcomes in HCC patients, highlighting the oncogenic role of BPTF in this context. In addition, considering previous reports showing that KD of BPTF attenuates HPSE expression,^12^ an enzyme critical for the cleavage of NCR ligands on the surface of tumor cells, we aimed to investigate whether this regulatory effect exists in HCC. Differential expression analysis of HPSE was performed based on the HCC patient cohort from The Cancer Genome Atlas database. The results showed that the expression of HPSE was significantly elevated in tumor tissues in comparison to adjacent normal tissues (Figure S1D). The immunoblotting results further confirmed the higher expression of HPSE in human HCC tumors (Figure S1E). Moreover, a positive correlation was observed between the expression of HPSE and BPTF in this HCC cohort (Figure S1F).

To elucidate the specific mechanism by which BPTF regulates HPSE expression, we performed RNA sequencing (RNA-seq) and RT-qPCR analyses on HCC cell line Huh7 that were treated with either BPTF small interfering RNA (siRNA) or a control siRNA. Treatment with BPTF siRNA to induce KD of BPTF resulted in a significant reduction in *HPSE* mRNA expression levels in Huh7 cells (Figures 1B and 1C), indicating that BPTF plays a regulatory role in controlling HPSE expression. Considering previous studies have identified the plant homeodomain finger of BPTF as a highly specialized methyl-lysine-binding domain for H3K4me3,^30^^,^^31^ which is an epigenetic modification marking the transcription start sites of nearly all active genes.^32^ This direct association between BPTF and this epigenetic signature suggests a potential regulatory mechanism by which BPTF may interpret H3K4me3 marks to modulate HPSE expression. Subsequently, cleavage under targets and tagmentation-(CUT&Tag) sequencing was performed to assess the presence of BPTF and H3K4me3 at the promoters of *HPSE* in Huh7 cells. Our analysis revealed a spatial overlap between the peaks of BPTF and H3K4me3 at the *HPSE* promoter region (Figure 1D), implying that both factors are likely involved in regulating HPSE gene expression. The direct interaction between BPTF and H3K4me3 in Huh7 cells was further confirmed through the immunoprecipitation (IP) experiment (Figure 1E). To investigate the co-enrichment pattern of BPTF and H3K4me3 at the *HPSE* promoter, a CUT&Tag assay followed by qPCR analysis was performed in Huh7 cells. The results unequivocally demonstrated the recruitment of BPTF to the *HPSE* promoter region (Figure 1F). Similarly, these findings also revealed a comparable enrichment profile for H3K4me3 at this specific promoter region (Figure 1G). Additionally, CUT&Tag assays followed by qPCR analyses were conducted to compare the level of H3K4me3 enrichment in the *HPSE* promoter region between BPTF wild-type (WT) and KD cells (Figure S2A). KD of BPTF did not result in down-regulation or up-regulation of the H3K4me3 level (Figures 1D and S2B), indicating that BPTF does not possess methylase or demethylase activity; rather, it functions as a reader for histone H3K4me3 to activate HPSE expression. Collectively, these results demonstrate how BPTF recognizes H3K4me3 at the *HPSE* promoter to facilitate its expression, indicating that the inhibition of BPTF could reduce HPSE levels in HCC cells.

### Design and synthesis of PROTACs targeting BPTF

**Figure 2:**
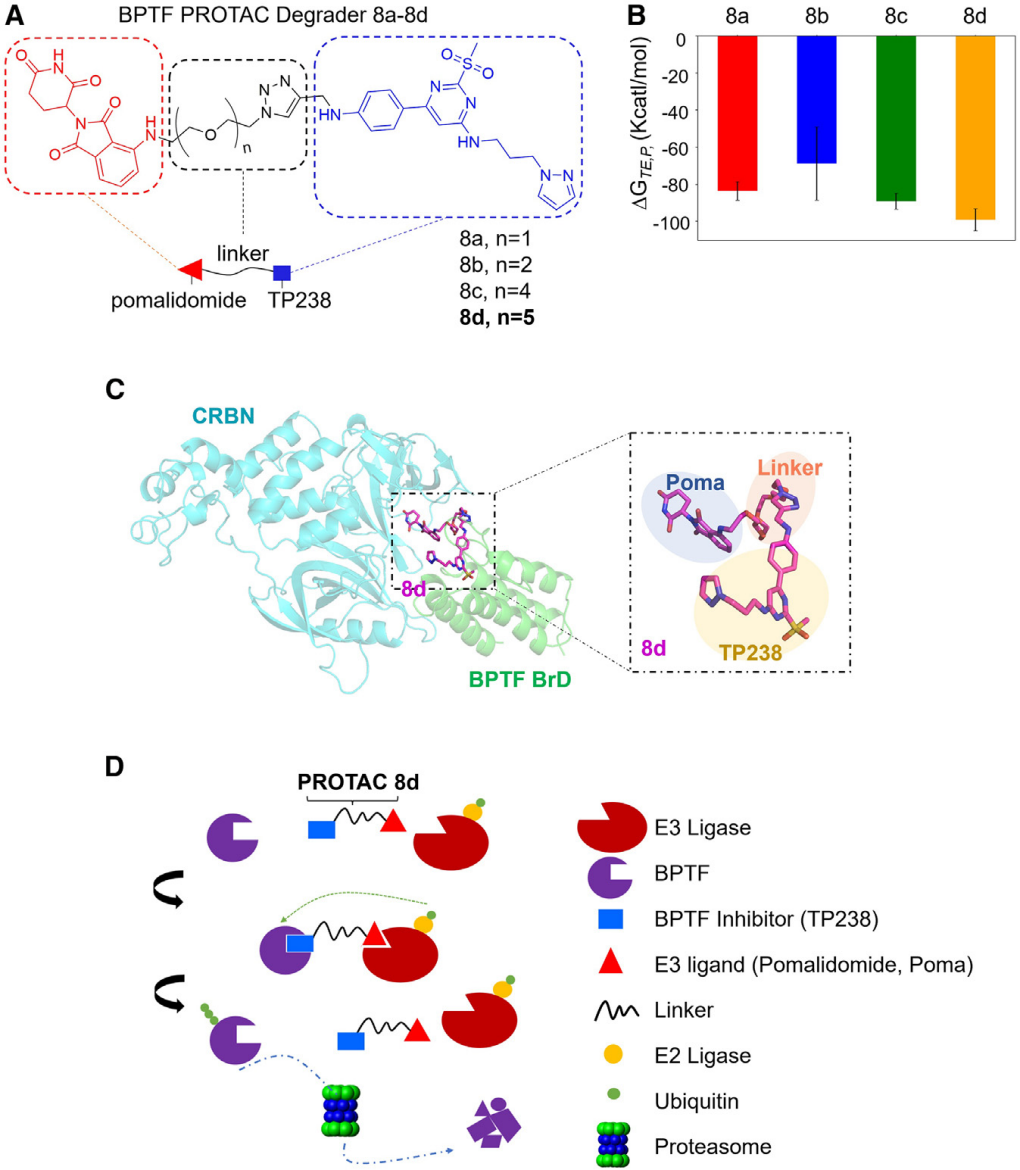
Design and synthesis of BPTF-targeting PROTACs (A) The chemical structure of BPTF PROTACs. The BPTF PROTAC degraders (**8a-d**) are designed with a structure that involves the conjugation of a BPTF inhibitor (TP238) to an E3 ligase binder (Poma) via linkers of varying PEG lengths. The TP238, E3 ligase binder, and linkers are highlighted in blue, red, and black respectively. (B) Binding energy of four different linker systems. T, E, and P symbolize the target protein, E3 ligase, and PROTAC molecule, respectively. The error bar was calculated as the standard deviation between different frames in a single molecular dynamics simulation system. (C) CRBN-PROTAC-BPTF ternary structure of the best-ranked **8d**, in which the chemical structure of PROTAC was highlighted. CRBN was shown cyan and BPTF was shown green. The highlighted PROTAC molecule was represented using multiple colors, with TP238 in yellow, Poma in blue, and linker in orange. (D) Scheme of the action model for the designed BPTF PROTAC degrader. The PROTAC degrader recruits E3 ligase to induce ubiquitination and subsequent proteasomal degradation of BPTF.

To achieve specific inhibition of BPTF while minimizing off-target interactions and completely abolishing its function, we used PROTAC technology to develop potent degraders that target BPTF. Consequently, four different degraders (**8a-d**) with varying linker lengths were synthesized by conjugating TP238, a potent inhibitor of BPTF bromodomain and also a promising candidate for the synthesis of BPTF degrader,^20^^,^^28^ with pomalidomide (Poma).^33^ The synthesis was achieved through classic click chemistry between compound **6** containing an alkyne group and Poma analogues **8a-d** containing an azido group (Figures 2A and S3). In contrast, a similar methodology was used to synthesize negative degradants **16** and **17** (Figures S4 and S5). Deactivated degrader **16** was synthesized by N-methylation of the glutarimide ring in Poma from compound **8d** to eliminate its binding affinity toward CRBN. Deactivated degrader **17** was obtained by substituting the methanesulfonate group with a methyl ester to obstruct the binding of compound **8d** with BPTF. All compounds were successfully validated (Figure S6).

Furthermore, considering the crucial role of the formation of the ternary complex between the target protein-PROTAC-E3 ligase in successful degradation of target proteins,^34^ we conducted molecular dynamics simulations lasting for 200 ns to predict the degradability of compounds **8a-d** by monitoring their respective binding free energy to their target proteins. Binding free energy refers to the change in energy that occurs when molecules form a complex and can either release or absorb energy during this process. It serves as a critical metric for evaluating the binding strength between candidates and their target proteins within these systems. As depicted in Figure 2B, compound **8d** exhibited significantly stronger binding affinity, highlighting its superior favorability within the solution environment. Additionally, trajectory clustering was performed to obtain representative conformations of the highest-performing ternary complex involving compound **8d** (Figure 2C). Theoretically, these PROTAC candidates are supposed to facilitate proximity between BPTF and E3 ligase, thereby leading to ubiquitination of BPTF followed by subsequent degradation through proteasomal machinery (Figure 2D).

### PROTACs exhibits selective degradation of BPTF in HCC cells

**Figure 3:**
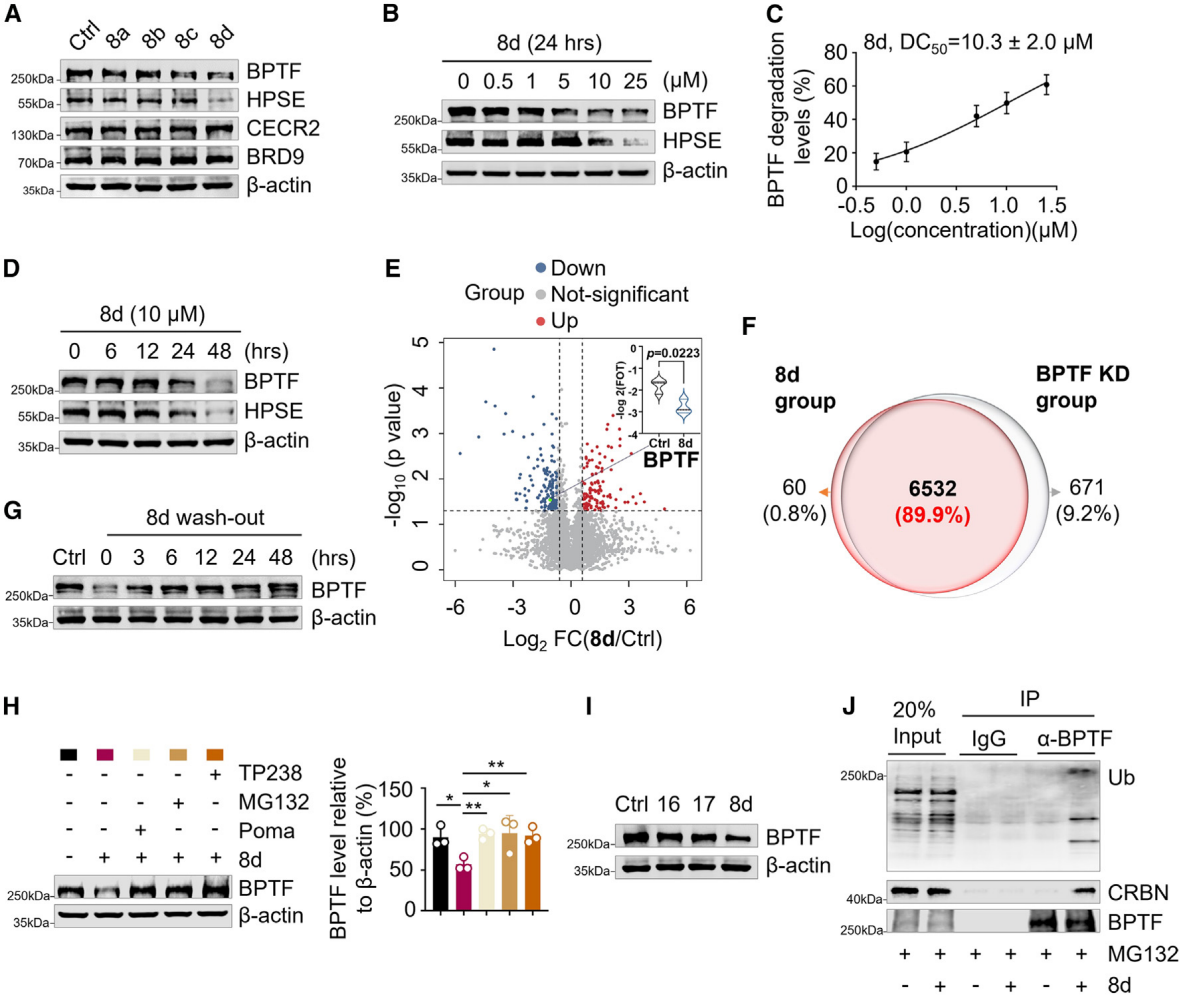
**8d** selectively degrades BPTF in a proteasome-dependent manner (A) Immunoblotting of BPTF, HPSE, CECR2 and BRD9 in the total cell lysates. The lysates were isolated from Huh7 cells treated with BPTF PROTAC degraders (**8a-d**) at 10 μM for 24 h, respectively. β-Actin was used as the loading control. The displayed image is representative of three biological replicates. (B) Immunoblotting of BPTF and HPSE in the total cell lysates. The lysates were isolated from Huh7 cells treated with **8d** at the indicated doses (0, 0.5, 1, 5, 10, and 25 μM) for 24 h β-actin was used as the loading control. The displayed image is representative of three biological replicates. (C) Degradation curves of BPTF in **8d**-treated Huh7 cells. The expression level of BPTF was normalized to the level of β-actin. *n* = 3. (D) Immunoblotting of BPTF and HPSE in the total cell lysates. The lysates were isolated from Huh7 cells treated with **8d** at 10 μM for the indicated times (0, 6, 12, 24, and 48 h). β-Actin was used as the loading control. The displayed image is representative of three biological replicates. (E) Volcanic plot illustrating the altered proteins between control and **8d**-treated Huh7 cells. The significantly downregulated and upregulated proteins are represented by blue and red dots, respectively. (Two-sided paired t test, *p* < 0.05, FC > 1.5). (F) Venn diagram showing the similarities and differences between **8d**-treated and BPTF KD Huh7 cell groups. (G) Immunoblotting of BPTF in the total cell lysates. The lysates were isolated from Huh7 cells that were pretreated with **8d** (10 μM) for 24 h followed by wash-out for the indicated times. β-Actin was used as the loading control. The displayed image is representative of three biological replicates. (H) Immunoblot analysis (left) and quantification (right, *n* = 3) of BPTF in the total cell lysates. The lysates were isolated from Huh7 cells that were respectively pretreated with Poma (1 μM), MG132 (1 μM), and TP238 (10 μM) for 1 h before **8d** treatment (10 μM for 24 h). β-Actin was used as the loading control. The displayed image is representative of three biological replicates. The data was presented as mean ± SD; ∗*p* < 0.05, ∗∗*p* < 0.01, ∗∗∗*p* < 0.001, ∗∗∗∗*p* < 0.0001; Unpaired t test. (I) Immunoblotting of BPTF in the total cell lysates. The lysates were isolated from Huh7 cells that were treated with the deactivated degraders **16**, **17** and PROTAC degrader **8d** (10 μM for 24 h), respectively. β-Actin was used as the loading control. The displayed image is representative of three biological replicates. (J) IP assay using anti-BPTF and IgG control to detect the endogenous association of BPTF with CRBN (E3 ligase) and the ubiquitination level of BPTF in Huh7 cells pretreated with MG132 (1 μM) for 3 h before **8d** treatment (10 μM for 24 h). The displayed image is representative of three biological replicates.

To further identify the most potent BPTF-targeting PROTAC degrader among the candidates **8a**-**8d**, we assessed their respective abilities to degrade both BPTF and its downstream effector, HPSE, in the HCC cell line Huh7. Consistent with our molecular dynamics simulations, immunoblotting analysis confirmed that compound **8d** outperforms compounds **8a-c** in terms of its capacity to degrade these target proteins (Figure 3A). Furthermore, compound **8d** exhibited no impact on CECR2 and BRD9 (Figures 3A and S7A), which have been previously reported as targets of TP238.^17^^,^^35^^,^^36^

To demonstrate that compound **8d** specifically targets the BPTF bromodomain rather than BRD9 or CECR2, both of which are also targeted by TP238, we conducted surface plasmon resonance (SPR) analysis. The results indicated a significant binding affinity between **8d** and the BPTF bromodomain (dissociation constant [*K*_d_] = 0.5629 μM), whereas the *K*_d_ for **8d** and BRD9 was 28.82 μM (Figures S7B and S7C). Furthermore, **8d** exhibited minimal affinity for the CECR2 bromodomain (Figure S7D), which is known to strongly bind to TP238 as a positive control. Our SPR data revealed a significantly higher binding affinity of **8d** for BPTF compared with CECR2 and BRD9. This enhanced affinity, in conjunction with potentially favorable ternary complex geometry and E3 ligase interactions, may contribute to the observed selectivity for BPTF degradation. This finding, along with other reports, highlights the potential of PROTAC technology to achieve selective degradation, even within a family of closely related proteins. Given **8d**’s superior performance, we selected **8d** as the lead candidate for further investigation.

The efficacy of compound **8d**-induced degradation of BPTF was subsequently evaluated. As shown in Figures 3B and 3C, a dose-dependent reduction with a half-maximal degradation (DC_50_) value of 10.3 ± 2.0 μM was observed in BPTF expression. Additionally, a time course experiment confirmed significant degradation of BPTF after exposure to compound **8d** for 24 h (Figure 3D). Consequently, the optimal conditions (10 μM for 24 h) were used for subsequent investigations involving treatment with compound **8d**.

The selectivity of **8d**’s degradative effect was further investigated by conducting proteomics analyses on Huh7 cells treated with either **8d** or BPTF siRNA. Within the group treated with **8d**, a total of 980 proteins exhibited reduced expression levels, out of which 173 showed a substantial decrease (>1.5-fold change [FC]; *p* < 0.05). Notably, BPTF was among the proteins significantly diminished by **8d** (Figure 3E). A comparative analysis between the groups treated with **8d** and BPTF KD revealed an impressive concurrence rate of 89.9% in terms of downregulated proteins (Figures 3F and S8A), thereby confirming that the effects of **8d** closely resemble those induced by BPTF siRNA. This high degree of overlap was further supported by correlation analysis (Figure S8B), providing additional evidence for the selective degradation facilitated by **8d**.

To investigate whether the degradation process of BPTF by **8d** is reversible, a wash-out experiment was conducted where Huh7 cells treated with **8d** were thoroughly rinsed to eliminate any residual compound. Subsequently, these cells were monitored for up to 48 h to observe the re-emergence of BPTF expression. The results demonstrated that, within 6 h after washout, BPTF began to recover (Figure 3G). This observation highlights the reversible nature of BPTF degradation mediated by **8d**.

To elucidate the underlying mechanism of **8d**-induced BPTF degradation, a series of co-treatment experiments were conducted in Huh7 cells by combining **8d** with specific inhibitors: Poma, which targets the CRBN E3 ligase; MG132, a potent proteasome inhibitor; and TP238, a direct BPTF inhibitor. The results demonstrated that the addition of Poma, MG132, and TP238 effectively reversed the **8d**-induced degradation of BPTF (Figure 3H), indicating the crucial role played by **8d**’s interaction with both BPTF and the CRBN E3 ligase in promoting BPTF degradation. Moreover, the effects of the two deactivated degraders, **16** and **17**, on BPTF degradation were evaluated in Huh7 cells. Neither compound **16** nor compound **17** could induce BPTF degradation (Figure 3I), further supporting the crucial role of the interaction between **8d**, BPTF, and the CRBN E3 ligase in promoting BPTF degradation. Furthermore, the ubiquitination of BPTF was significantly increased in Huh7 cells treated with **8d** (Figure 3J), indicating that **8d**-induced BPTF downregulation occurs through ubiquitin-mediated degradation. These findings substantiate the proposition that, through direct recruitment of CRBN, **8d** facilitates the proteasome-dependent selective degradation of BPTF.

### PROTAC treatment enhances the effector function of NK cells toward HCC cells

**Figure 4:**
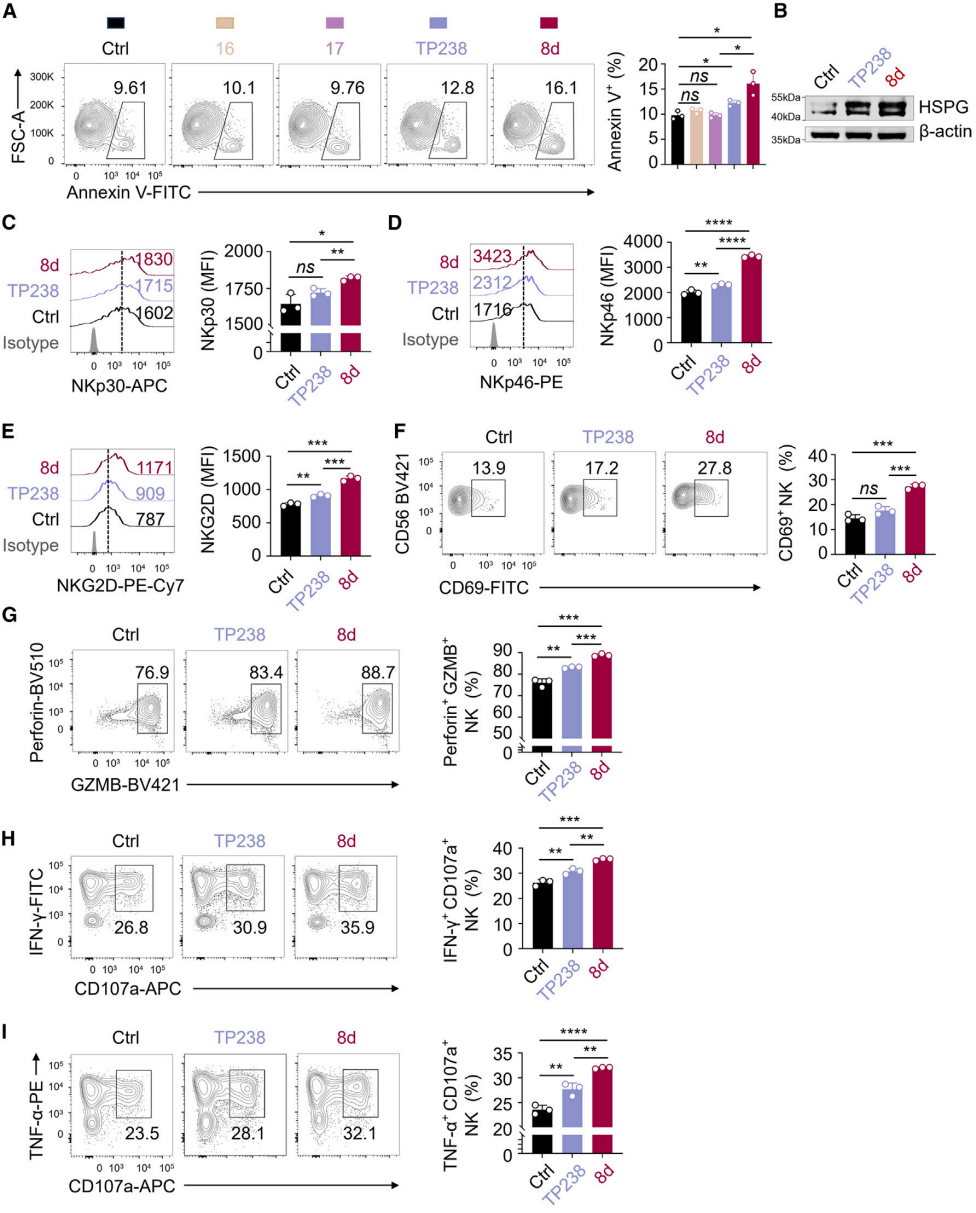
The effector function of human primary NK cells toward Huh7 cells is enhanced by **8d** treatment (A) Flow cytometry analysis (left) and quantification (right, *n* = 3) showing the percentage of Annexin V^+^ Huh7 cells (target cells) in a co-culture with human primary NK cells (effector cells) for 4 h at an effector/target ratio of 1:20. The control Huh7 cells, **16**-treated Huh7 cells, **17**-treated Huh7 cells, TP238-treated Huh7 cells and **8d**-treated Huh7 cells were included in the experiment. (B) Immunoblotting of HSPG in the total cell lysates. The lysates were obtained from Huh7 cells treated with TP238 (10 μM) or **8d** (10 μM) for 24 h β-actin served as the loading control. The presented image is representative of three independent experiments. (C–E) Flow cytometry analysis (left) and quantification (right, *n* = 3) showing the expression of NKp30 (C), NKp46 (D), and NKG2D (E) in primary human NK cells. The NK cells were co-cultured with control Huh7 cells, TP238-treated Huh7cells, and **8d**-treated Huh7 cells for a duration of 4 h. (F–I) Flow cytometry analysis (left) and quantification (right, *n* = 3) showing the proportion of CD69^+^ NK cells (F), Perforin^+^ GZMB^+^ NK cells (G), IFN-γ^+^ CD107a^+^ NK cells (H), and TNF-α^+^ CD107a^+^ NK cells (I) within the total population of primary human NK cells. The NK cells were co-cultured with control Huh7 cells, TP238-treated Huh7cells and **8d**-treated Huh7 cells for a duration of 4 h. The data in (A) and (C–I) were presented as mean ± SD; ∗*p* < 0.05, ∗∗*p* < 0.01, ∗∗∗*p* < 0.001, ∗∗∗∗*p* < 0.0001; unpaired t test.

To determine whether the reduction of BPTF increases the susceptibility of HCC cells to NK cell-mediated cytotoxicity, we evaluated the percentage of apoptotic HCC cells as a measure of NK cell cytotoxicity. Human primary NK cells showed a significantly enhanced cytotoxicity against BPTF-KD Huh7 cells compared with control cells (Figure S9), indicating that the inhibition of BPTF in HCC cells effectively activates NK cell responses. To simulate the tumor microenvironment where tumor cells are predominant,^37^ we set the effector/target ratio to 1/20 for subsequent co-culture of human primary NK cells with Huh7 cells. As depicted in Figure 4A, primary NK cells exhibited a significant enhanced cytotoxicity against Huh7 cells pretreated with **8d** compared with those pretreated with control or TP238, indicating the superior efficacy of **8d** over TP238 in augmenting NK cell-mediated cytotoxicity. This indicates that PROTAC acts as an alternative strategy capable of fully suppressing the function of BPTF, rather than solely targeting BPTF bromodomains, which may not completely suppress all functions associated with other domains. Moreover, treating Huh7 cells with the two deactivated degraders, **16** and **17**, failed to induce the cytotoxicity of primary NK cells (Figure 4A), further supporting the effect of **8d** in activating NK cell response. To exclude the impact of **8d** toxicity, we evaluated the proportions of apoptotic Huh7 cells or primary NK cells treated exclusively with **8d**. Interestingly, **8d** did not affect the cell viability of either cell (Figures S10A and S10B), indicating that apoptosis induction in Huh7 cells is not caused by **8d** itself. To investigate whether the degradation of BPTF by **8d** treatment leads to decreased HPSE expression and increased abundance of HSPGs (NCR ligands) on HCC cells, we measured the levels of HSPGs using an HS antibody for immunoblotting.^12^ The immunoblotting results showed that two specific bands, an approximately 45-kDa band and an approximately 50-kDa band, had significantly increased in **8d**-treated Huh7 cells compared with the control or TP238-treated cells (Figure 4B), while they did not change in the deactivated degraders **16 or 17** treatment group (Figure S11). These findings indicate that **8d** treatment increases the levels of HSPGs on HCC cells.

To further investigate the potential of **8d** treatment in activating NK cells, we assessed the expression of activating receptors on NK cells. As depicted in Figures 4C–4F, when human primary NK cells were co-cultured with **8d**-pretreated Huh7 cells, the levels of activating receptors, including NCRs (NKp30, NKp46), NKG2D, and CD69 were significantly elevated compared with those co-cultured with control or TP238-pretreated HCC cells. However, when primary NK cells were treated with **8d** in the absence of HCC cells, there was no noticeable effect on the upregulation of NK cell-activating receptors (Figures S12A–S12D), ruling out a direct effect of **8d** on NK cell activation. Moreover, the proportions of poly-functional effector NK cells, including perforin^+^granzyme B (GZMB)^+^, interferon (IFN)-γ^+^CD107a^+^ and tumor necrosis factor (TNF)-α^+^CD107a^+^ NK cells, were significantly increased in primary NK cells co-cultured with **8d**-pretreated HCC cells compared with those co-cultured with control or TP238-pretreated HCC cells (Figures 4G–4I). These findings support that **8d** treatment enhances the effector function of NK cells toward HCC cells.

### PROTAC treatment enhances the effector function of NK cells against primary HCC cells isolated from tumor tissues of HCC patients

**Figure 5:**
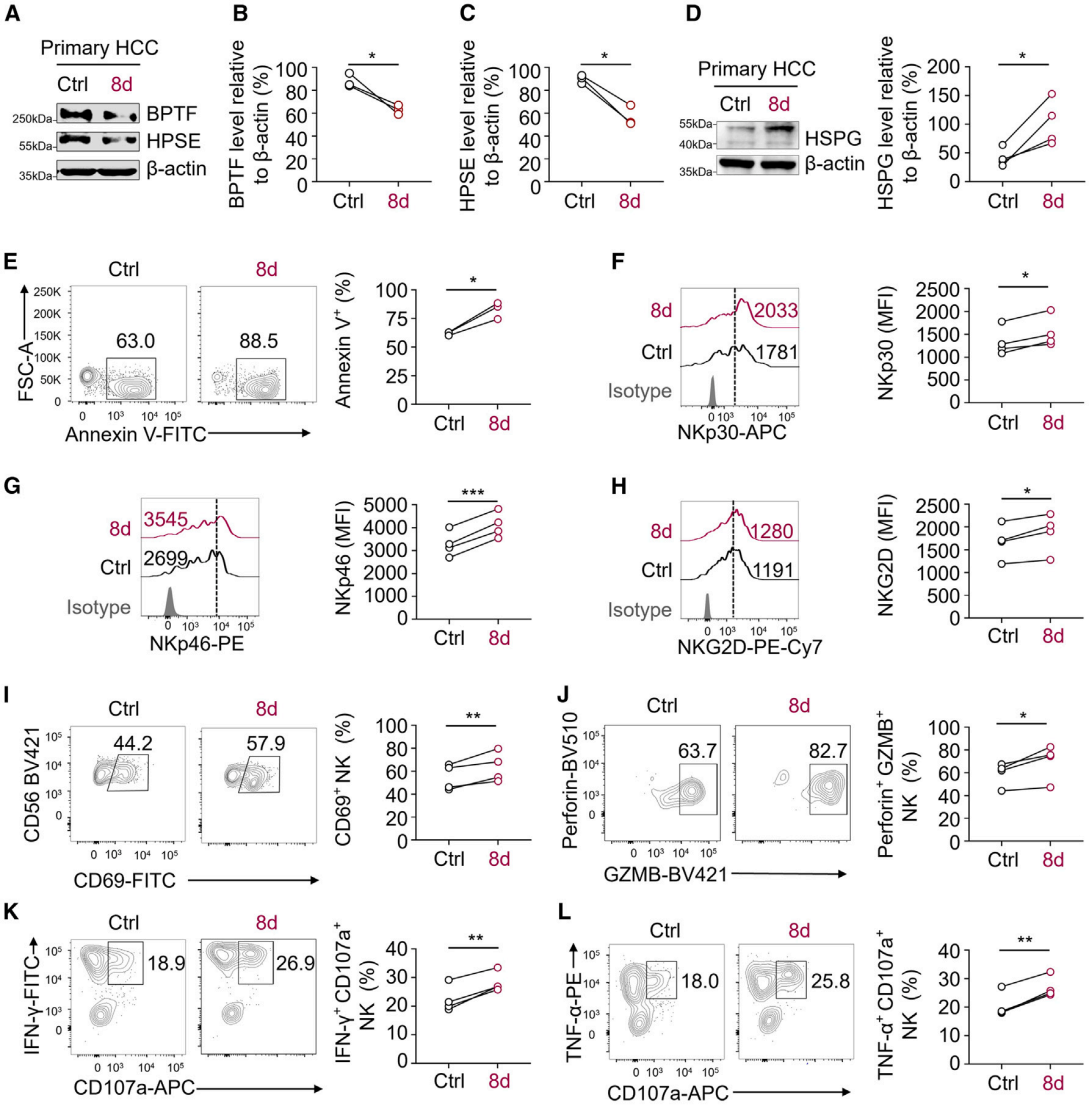
The effector function of primary human NK cells toward primary HCC cells isolated from the tumor tissue of HCC patients is enhanced by **8d** treatment (A) Immunoblotting of the expression of BPTF and HPSE in the lysates extracted from primary HCC cells isolated from the tumor tissue of HCC patients. The primary HCC cells were treated with or without **8d** (10 μM for 24 h) before lysate extraction. β-Actin was used as the loading control. The presented image is representative of three biological replicates. (B and C) Quantification of the expression level of BPTF (B) and HPSE (C) depicted in (A). The expression levels of BPTF and HPSE were normalized to the levels of β-actin. *n* = 3. (D) Immunoblotting of the expression of HSPG (left) and quantification (right, *n* = 4) in the lysates extracted from primary HCC cells isolated from tumor tissue of HCC patients. The primary HCC cells were subjected to treatment with or without **8d** (10 μM for 24 h) before lysate extraction. β-Actin was used as the loading control. The image presented is representative of four biological replicates. (E) Flow cytometry analysis (left) and quantification (right, *n* = 3) showing the percentage of AnnexinV^+^ primary HCC cells (target cells) in a co-culture with primary human NK cells (effector cells) for 4 h, with a ratio of effector/target at 1:20. The control and **8d**-treated primary HCC cells were included in the experiment. (F–H) Flow cytometry analysis (left) and quantification (right, *n* = 4) showing the expression of NKp30 (F), NKp46 (G), and NKG2D (H) on primary human NK cells following a 4-h co-culture with either control or **8d**-treated primary HCC cells. (I–L) Flow cytometry analysis (left) and quantification (right, *n* = 4) showing demonstrated the proportion of CD69^+^ NK cells (I), Perforin^+^GZMB^+^ NK cells (J), IFN-γ^+^CD107a^+^ NK cells (K), and TNF-α^+^CD107a^+^ NK cells (L) within the total population of primary human NK cells after a 4-h co-culture with either control or **8d**-treated primary HCC cells. The data in (B–L) are presented as mean ± SD; ∗*p* < 0.05, ∗∗*p* < 0.01, ∗∗∗*p* < 0.001, ∗∗∗∗*p* < 0.0001; paired t test.

Afterward, we proceeded to validate whether the degradation of BPTF by PROTAC **8d** could enhance the susceptibility of primary HCC cells, isolated from HCC patients’ tumor tissues, to NK cell-mediated cytotoxicity. First, we examined the effectiveness of **8d**-induced BPTF degradation in primary HCC cells. The immunoblotting results showed a significant reduction in the expression levels of both BPTF and HPSE in primary HCC cells treated with **8d** (Figures 5A–5C). Additionally, the levels of HSPGs were found to be elevated in primary HCC cells treated with **8d** (Figure 5D). Furthermore, the proportion of apoptotic primary HCC cells co-cultured with human primary NK cells were quantified. As depicted in Figure 5E, primary NK cells exhibited significantly elevated cytotoxicity toward **8d**-pretreated primary HCC cells compared with control primary HCC cells, indicating that **8d** treatment effectively enhances NK cell-mediated cytotoxicity against primary HCC cells. Consistent with results from HCC cell line experiments, treatment with **8d** alone did not significantly affect the viability of primary HCC cells (Figure S13), indicating that apoptosis induction in these cells is not caused by **8d** itself.

After that, we investigated whether **8d** treatment could activate and enhance the effector function of NK cells. Compared with primary NK cells co-cultured with control primary HCC cells, we observed increased expression of activating receptors (NKp30, NKp46, and NKG2D) and CD69 on primary human NK cells co-cultured with **8d**-pretreated primary HCC cells (Figures 5F–5I). Moreover, the proportion of poly-functional effector NK cells, including those expressing perforin^+^ GZMB^+^, IFN-γ^+^CD107a^+^ and TNF-α^+^CD107a^+^, was significantly increased in primary NK cells co-cultured with **8d**-pretreated primary HCC cells (Figures 5J–5L). These findings demonstrate that treatment with **8d** enhances the effector function of NK cells against primary HCC cells isolated from tumor tissues of patients, indicating the potential for clinical application of **8d** as an immunotherapeutic agent.

**Figure 6:**
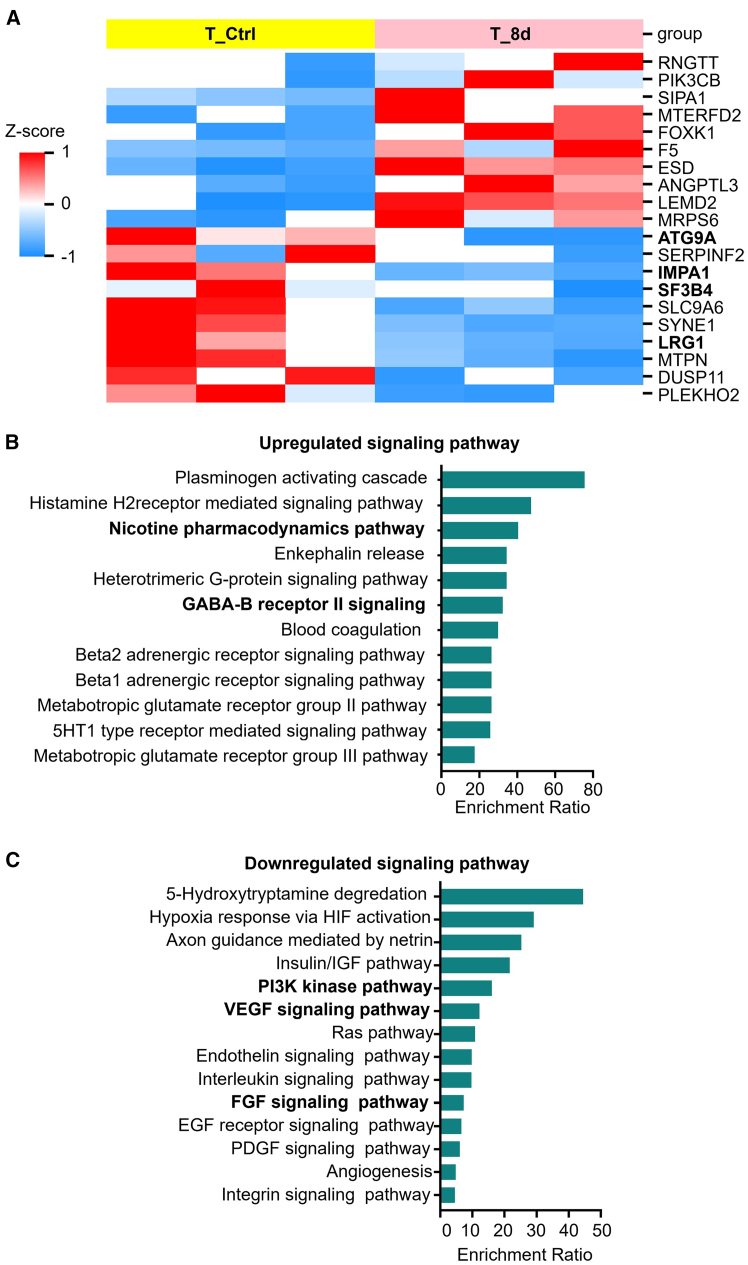
Proteomic profiling reveals the effect of **8d** on primary HCC cells isolated from tumor tissues of HCC patients (A) Heatmap displays the normalized protein expression levels in primary HCC cells treated with control (yellow) or **8d** (pink), (*n* = 3). Each column represents one individual sample. (B and C) The pathway enrichment analysis comparing control and **8d**-treated primary HCC cells, which is enriched with up-regulated (B) or down-regulated (C) proteins. EGF, epidermal growth factor; FGF, fibroblast growth factor; HIF, hypoxia-inducible growth factor; IGF, insulin-like growth factor; PDGF, platelet-derived growth factor; VEGF, vascular endothelial growth factor.

Next, we conducted proteomic profiling to explore the effect of **8d** on primary HCC cells isolated from tumor tissues of HCC patients. Compared with the control cells, the tumor-promoting proteins such as ATG9A,^38^ IMPA1,^39^ SF3B4,^40^^,^^41^ and LRG1^42^^,^^43^ were significantly downregulated in primary HCC cells treated with **8d** (Figure 6A). Furthermore, functional enrichment analyses indicated significant changes in signaling pathways that regulate cancer cell metabolism and development in primary HCC cells treated with **8d** compared with control cells. Specifically, a multitude of tumor suppressor pathways have been upregulated, including the nicotine pharmacodynamics pathway,^44^ and gamma aminobutyric acid-B receptor II signaling pathway (Figure 6B).^45^^,^^46^ Conversely, numerous pathways involved in promoting cancer development have been downregulated, such as the PI3K kinase pathway,^47^ vascular endothelial growth factor signaling pathway,^48^ fibroblast growth factor signaling pathway,^49^ and so forth (Figure 6C). These findings suggest that **8d** exhibits potential in reducing the tumorigenic properties of HCC cells, thereby further supporting its promise as an immunotherapeutic agent for targeting HCC.

### PROTAC treatment leads to BPTF degradation and inhibits the *in vivo* outgrowth of HCC

**Figure 7:**
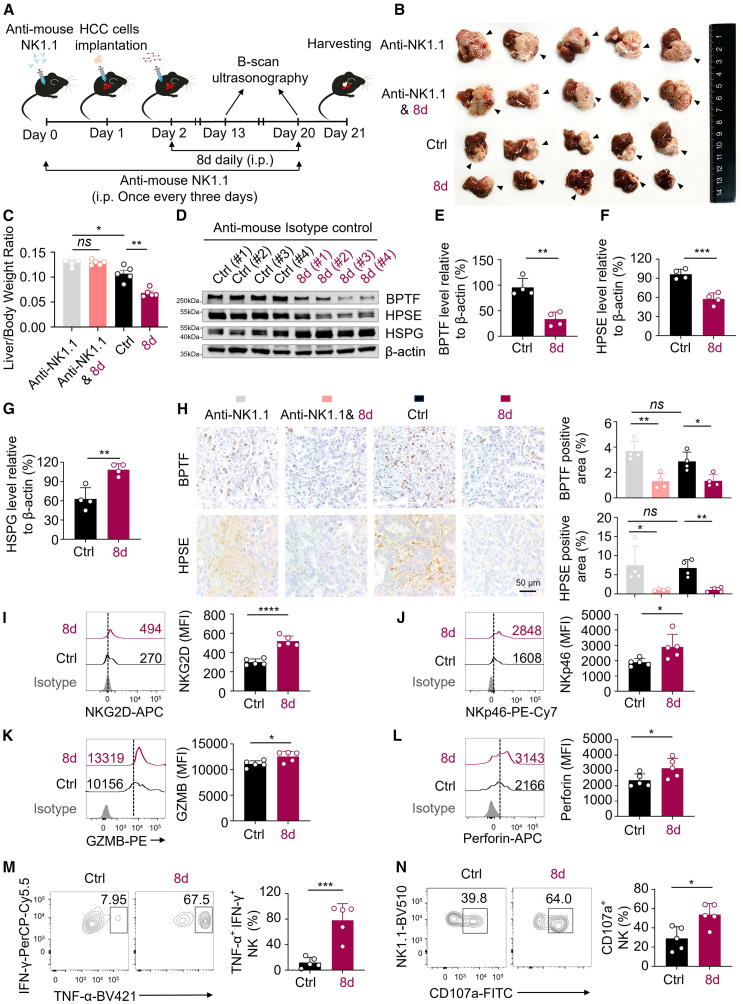
**8d** treatment prevents HCC outgrowth *in vivo* (A) Treatment schedule of **8d** in the orthotopic HCC mouse model, both in the presence of NK cells and after their depletion by applying anti-mouse NK1.1 antibody. (B) Representative images of HCC on day 21 from four different groups: HCC mice with NK cell depletion treated with vehicle (first row), HCC mice with NK cell depletion treated with **8d** (second row), control HCC mice (third row), and HCC mice treated with **8d** (fourth row). *n* = 5 for each group. The arrowheads indicate the area where the tumor is growing. (C) Quantification of liver/body weight ratio in mice from the four groups shown in (B) at autopsy on day 21. *n* = 5. (D–G) Immunoblot analysis and quantification (*n* = 4) of the expression of BPTF (D and E), HPSE (D and F), and HSPG (D and G) in the lysates extracted from the liver tissues of HCC mice in the control and **8d** groups. β-Actin was used as the loading control. (H) Representative images (left) and quantification (right, *n* = 4) of immunohistochemistry (IHC) staining of BPTF and HPSE in the liver tissue sections of HCC mice from the four groups shown in B at autopsy on day 21. Scale bars, 50 μm. I-N. Flow cytometry analysis (left) and quantification (right, *n* = 5) of the expression of NKG2D (I), NKp46 (J), GZMB (K), Perforin (L), IFN-γ^+^ TNF-α^+^ (M), and CD107a^+^ (N) on NK cells isolated from the liver tissues of HCC mice treated with control or **8d**. The data in (C) and (E–N) are presented as mean ± SD; ∗*p* < 0.05, ∗∗*p* < 0.01, ∗∗∗*p* < 0.001, ∗∗∗∗*p* < 0.0001; unpaired t test.

To further investigate the potential inhibitory effects of PROTAC **8d** treatment on HCC growth *in vivo*, we established an orthotopic HCC model by injecting a murine HCC cell line^50^ into mice. The HCC mice were administered a daily dose of **8d** at 10 mg/kg for 20 days, with or without NK cells mediated by anti-mouse NK1.1 antibody (Figures 7A and S14A). Notably, treatment with **8d** resulted in better therapeutic effects on HCC in comparison to the control group, as shown by decreased liver/body weight ratios and increased survival rates (Figures 7B, 7C, and S14B). B-mode ultrasound images taken on day 13 and day 20 after HCC cell implantation showed a smaller focal area in the **8d**-treated group (Figure S14C). In contrast with the control group, the depletion of NK cells mediated by anti-mouse NK1.1 antibody aggravated HCC outgrowth (Figures 7B, 7C, S14B, and S14C), underlining the crucial role of NK cells in controlling HCC progression. Significantly, compared with the group treated with **8d** alone, the depletion of NK cells eliminated **8d**’s inhibitory effect on HCC outgrowth (Figures 7B, 7C, S14B, and S14C), suggesting that **8d** inhibits HCC growth via NK cells. Moreover, a human hepatoma model was established in NCG mice, which lack functional/mature T, B, and NK cells because of triple immunodeficiency, with luciferase-labeled Huh7 cells (Figure S15A). Two days later, human primary NK cells were adoptively transferred into these mice. Treatment with **8d** effectively controlled tumor growth as opposed to the control group (Figures S15B and S15C), further confirming that **8d** inhibits HCC growth through NK cells *in vivo*. Furthermore, compared with the control group, the administration of **8d** did not result in any significant differences in body weight or kidney and spleen weights (Figures S16A–S16C). Also, it did not cause any pathological damage to the kidneys or spleens (Figure S16D), regardless of whether NK cells were depleted or not. These results indicate minimal *in vivo* toxicity levels.

In line with aforementioned results, **8d** treatment did lead to a decrease in BPTF and HPSE expression (Figures 7D–7F and 7H), consequently resulting in an increase of HSPG level within the hepatic tumor tissue of mice (Figures 7D and 7G). In hepatic tumor tissue of mice, the down-regulation of BPTF and HPSE, and the up-regulation of HSPG induced by **8d** were independent of NK cells, since similar results were observed in the NK cell-depleted group (Figures 7H and S17A–S17D). Moreover, in HCC mice treated with **8d**, intra-tumoral NK cells were activated, with increased levels of NKG2D, NKp46, GZMB, and perforin (Figures 7I–7L). Furthermore, the frequency of poly-functional effector NK cells, which are characterized by IFN-γ^+^, TNF-α^+^, and CD107a^+^ expression, significantly increased (Figures 7M and 7N). The findings suggest that the degradation of BPTF induced by **8d** hinders the progression of HCC by enhancing the *in vivo* antitumor activity of NK cells, indicating that a therapeutic strategy targeting BPTF degradation using PROTACs holds promise for halting HCC progression.

## Discussion

Our proteomics analysis suggests that BPTF represents a promising immunotherapeutic target in the treatment of HCC. Moreover, we demonstrate that BPTF functions as a reader for the histone mark H3K4me3 to initiate the expression of HPSE. This subsequently impairs NK cell recognition of HCC cells. Furthermore, we present the design and characterization of compound **8d** as a selective PROTAC specifically targeting BPTF. Importantly, the degradation induced by **8d** effectively increases the susceptibility of primary HCC cells isolated from tumor tissues to NK cell-mediated cytotoxicity and inhibits HCC outgrowth *in vivo*. Our study demonstrates that using a BPTF-degrading PROTAC enhances NK cell activity against HCC.

The significance of our findings is manifold. First, it expands the therapeutic potential of PROTAC degraders beyond conventional cancer treatments to the field of cancer immunotherapy, particularly enhancing the efficacy of NK cell antitumor responses. Traditional PROTACs have been engineered to target and eliminate oncoproteins that are overexpressed, hyperactive, or mutated, thereby contributing to cancer progression and ultimately inducing cancer cell death.^51^ In contrast, our developed PROTAC **8d** represents the BPTF-targeting PROTAC that exhibits minimal toxicity toward HCC cells or NK cells, setting itself apart from conventional PROTACs. Importantly, degradation of BPTF induced by **8d** leads to increased levels of NCR ligands on HCC cells, rendering them more susceptible to NK cell-mediated cytotoxicity. Our findings introduce novel opportunities for using PROTAC technology in the realm of cancer immunotherapy.

Second, BPTF emerges as a promising immunotherapeutic target for HCC treatment. Acknowledged as a pivotal transcription factor and epigenetic regulator,^17^^,^^20^^,^^30^ BPTF has captured our attention due to its role in modulating NK cell-mediated antitumor responses.^12^ Proteomic analysis of 159 HCC patients has revealed a positive correlation between BPTF expression and the progression of HCC. Mechanistically, BPTF functions as a reader of histone H3K4me3 marks, initiating the expression of HPSE, which impairs NK cell recognition by altering the abundance of NCR ligands on HCC cells. Moreover, **8d-**induced degradation of BPTF enhances the efficacy of NK cells against HCC. Collectively, our findings position BPTF as an innovative candidate for immunotherapy in treating HCC.

Third, our findings provide compelling evidence supporting the application of PROTAC degraders as a potential strategy to enhance the efficacy of NK cell antitumor responses. NK cell-based immunotherapy has emerged as a promising and effective treatment modality for patients with advanced cancers,^8^ but their antitumor efficacy is often compromised due to continuous exposure to tumor antigens within the tumor microenvironment, leading to NK cell exhaustion.^52^ Current interventions, such as immune checkpoint inhibitors or antibodies, have demonstrated partial success in rejuvenating exhausted NK cells^53^; however, there remains an unmet need for innovative approaches that can further augment the antitumor activity of NK cells. In this study, we developed a BPTF-targeting PROTAC that not only effectively enhances the effector function of NK cells against primary HCC cells isolated from tumor tissues of HCC patients but also inhibits HCC outgrowth *in vivo*. Therefore, our findings present a novel chemical biological approach using PROTACs to enhance the efficacy of NK cell-based tumor immunosurveillance and offer potential clinical benefits for patients with advanced cancers.

Since in our investigation a concentration of 10 μM was used for PROTAC **8d**, further study will be carried out to optimize its chemical structure. The aim is to determine the optimal dosage by improving the bioavailability of PROTAC **8d** through the optimization of the PEG linker. William C. K. Pomerantz and his colleagues have devoted significant effort to this field, leading to the successful development of PROTACs that target CECR2 or BPTF for degradation.^28^ We appreciate their dedication and contributions to advancing this important area of research. Moreover, further research will evaluate whether the BPTF-specific PROTAC degrader could enhance NK cell-mediated antitumor efficacy against other malignancies with significant BPTF overexpression, such as lung cancer and breast cancer.

## Materials and methods

### Synthesis of BPTF PROTAC of **8a-d**

A mixture of compound 1 (2.3 g, 12.8 mM), compound 2 (3.7 g, 25.6 mM) and Cs_2_CO_3_ (12.5 g, 38.4 mM) in DMF (60 mL) was stirred at 70°C overnight. The reaction mixture was diluted with water, extracted with ethyl acetate (3×), dried (Na_2_SO_4_), and concentrated. The product was purified by column chromatography to product 3 (1.6 g, 44% yield) as a light yellow oil.

To a solution of compound 3 (1.6 g, 5.6 mM) in DCM was added *m*-CPBA (2.9 g, 16.7 mM) at 0°C. The mixture was stirred at rt for 2 h under N_2_ atmosphere before being diluted with DCM. The mixture was washed with sodium sulfate (sat. aq.), a NaOH solution (1 M), and brine and then dried over Na_2_SO_4_. The organic phase was filtered, concentrated, and purified by column chromatography to give compound 4 (1.6 g, 91% yield) as the desired product.

To a solution of compound 4 (1.6 g, 5.1 mM) in 1, 4-dioxane/H_2_O (5:4) were added 4-aminophenylboronic acid pinacol ester (1.7 g, 7.6 mM), K_2_CO_3_ (2.1 g, 15.2 mM), and Pd (PPh3) 4 (0.3 g, 0.25 mM). The mixture was purged with N_2_ and stirred at 80°C overnight. The mixture was filtered and purified by column chromatography give the desired product 5 (258.3 mg, 14% yield).

A mixture of 5 (300 mg, 0.8 mM), 3-Bromopropyne (288 mg, 2.4 mM) Cs_2_CO_3_ (788 mg, 2.4 mM), and KI (13.28 mg, 0.08 mM) was added in anhydrous DMF. After being stirred overnight at room temperature, the mixture was quenched with water and extracted with ethyl acetate. The organic layer was washed with water and brine, dried over anhydrous sodium sulfate, filtered, and then concentrated under reduced pressure. The residue was purified by silica gel chromatography to give pure compound 6 (113.8 mg, 34% yield).

Compound 6 (35 mg, 0.09 mM), compound 7d (60 mg, 0.1 mM), CuSO_4_ (1.5 mg, 0.009 mM), and TBTA (4.8 mg, 0.009 mM) were dissolved in DMF (800 μL). Then the sodium ascorbate (17.9 mg, 0.09 mM, dissolved in water) was added in the reaction. After the mixture was stirred overnight at room temperature, the reaction was quenched by water and the mixture was washed with saturated aqueous NaCl, extracted by EtOAc. The organic layer was dried over anhydrous Na_2_SO_4_, and evaporated in vacuum to afford the crude product, which was further purified by silica gel chromatography to give compound **8d** (35 mg, 40% yield). The same procedures were used for synthesis of **8a-c**: 4-((17-(4-(((4-(6-((3-(1H-pyrazol-1-yl)propyl)amino)-2-(methylsulfonyl)pyrimidin-4-yl)phenyl)amino)methyl)-1H-1,2,3-triazol-1-yl)-3,6,9,12,15 pentaoxaheptadecyl) amino)-2-(2,6-dioxopiperidin-3-yl) isoindoline-1,3-dione (**8d**): 1H NMR (600 MHz, DMSO-d6): δ ppm 11.08 (s, 1H), 7.95 (s, 3H), 7.78 (s, 1H), 7.75 (d, J = 2.0 Hz, 1H), 7.63–7.54 (m, 2H), 7.44 (s, 1H), 7.12 (d, J = 8.6 Hz, 1H), 7.03 (d, J = 7.0 Hz, 1H), 6.85 (s, 1H), 6.75 (d, J = 8.8 Hz, 2H), 6.58 (s, 1H), 6.23 (s, 1H), 5.04 (dd, J = 12.8, 5.4 Hz, 1H), 4.48 (t, J = 5.2 Hz, 2H), 4.37 (s, 2H), 4.20 (s, 2H), 3.54–3.53 (m, 2H), 3.51–3.50 (m, 2H), 3.49–3.40 (m, 18H), 3.32 (s, 2H), 3.29 (s, 3H), 2.91–2.84 (m, 1H), 2.62–2.52 (m, 2H), 2.07–2.03 (m, 2H), 2.03–2.00 (m, 1H). 13C NMR (150 MHz, DMSO-d6): δ ppm 173.3, 170.6, 169.5, 167.8, 164.0, 158.9, 158.6, 146.9, 145.5, 139.1, 136.7, 132.6, 130.4, 128.9, 128.3, 123.7, 118.0, 117.0, 115.2, 112.6, 111.2, 109.8, 105.5, 70.34, 70.29, 70.26, 70.14, 70.09, 69.4, 69.3, 49.9, 49.1, 42.2, 39.2, 38.7, 31.5, 22.7.HR-MS (ESI) m/z: calcd forC45H56O11N12NaS [M + Na]+, 995.3804; found, 995.3803. High-performance liquid chromatography purity was 97%.

### Synthesis of deactivated degrader of 16–17

To a solution of 9 (200 mg, 0.7 mM) in DMF (5 mL) were added Cs_2_CO_3_ (351 mg, 1.1 mM) and CH3I (153 mg, 1.1 mM) at room temperature. The resulting mixture was stirred overnight. The reaction was quenched by H_2_O, then extracted with ethyl acetate. The combined organic layers were washed with brine, dried over anhydrous sodium sulfate, and concentrated under reduced pressure. The residue was purified by silica gel flash column chromatography to afford compound 10 (174 mg, 87% yield).

A mixture of 10 (100 mg, 0.3 mM), 17-Azido-3, 6, 9, 12, 15-pentaoxaheptadecan-1-amine (100 mg, 0.3 mM), and DIEA (88 mg, 0.7 mM) in dry DMF (5 mL) was stirred in a round-bottom flask for 6 h at 90°C. The reaction was quenched by water and the mixture was washed once with saturated aqueous NaCl, extracted by EtOAc. The organic layer was dried over anhydrous Na_2_SO_4_, and evaporated in vacuum to afford the crude product, which was further purified by silica gel column chromatography to give compound 11 (122.5 mg, 40% yield).

A mixture of compound 12 (500 mg, 3.1 mM), compound 2 (903 mg, 6.3 mM), and Cs_2_CO_3_ (3 g, 9.4 mM) in DMF (20 mL) was stirred at 70°C overnight. The reaction mixture was diluted with water, extracted with ethyl acetate (3×), dried (Na_2_SO_4_), and concentrated. The product was purified by column chromatography to product 13 (240 mg, 29% yield).

To a solution of compound 13 (240 mg, 0.9 mM) in 1, 4-dioxane/H_2_O (5:4) were added 4-aminophenylboronic acid pinacol ester (280 mg, 1.3 mM), K_2_CO_3_ (354.5 mg, 2.6 mM), and Pd (PPh3) 4 (49.7 mg, 0.04 mM). The mixture was purged with N_2_ and stirred at 80°C overnight. The mixture was filtered and purified by column chromatography give the desired product 14 (150 mg, 54% yield).

A mixture of 14 (150 mg, 0.5mmol), 3-bromopropyne (164 mg, 1.4 mM), Cs_2_CO_3_ (450 mg, 1.4 mM) ,and KI (8.3 mg, 0.05 mM) was added in anhydrous DMF. After stirring overnight at room temperature, the mixture was quenched with water and extracted with ethyl acetate. The organic layer was washed with water and brine, dried over anhydrous sodium sulfate, filtered and then concentrated under reduced pressure. The residue was purified by silica gel chromatography to give pure compound 15 (50 mg, 30% yield).

Compound 6 (20 mg, 0.05 mM), compound 11 (35 mg, 0.06 mM), CuSO_4_ (1 mg, 0.005 mM), and TBTA (3 mg, 0.005 mM) were dissolved in DMF (800 μL). Then the sodium ascorbate (10 mg, 0.05 mM, dissolved in water) was added in the reaction. After the mixture was stirred overnight at room temperature, the reaction was quenched by water and the mixture was washed with saturated aqueous NaCl, extracted by EtOAc. The organic layer was dried over anhydrous Na_2_SO_4_, and evaporated in vacuum to afford the crude product, which was further purified by silica gel chromatography to give compound 16 (10.6 mg, 21% yield). The same procedures were used for synthesis of compound 17.

### Structure preparation

To construct the ternary complex CRBN-PROTAC-BPTF, we adopted the method of warhead alignment reported in previous literature.^34^ We initiated the process with the crystallographic structures of the ligand-bound CRBN (PDB ID: 4CI1) and ligand-bound BPTF (PDB ID: 7KDZ). Additionally, we utilized the reported PROTAC ternary complex crystal structure (PDB ID: 6BOY) as our template. This structure involves CRBN as the E3 ligase and BRD4 BD1 as the target protein—a domain shared with BPTF. Initially, we superimposed two warhead structures onto 6BOY and manually positioned the linker building blocks (e.g., −CH2OCH2−) between the two warheads on the protein surface. Subsequently, the leap and sander modules of Amber22 software package facilitated the linkage of these building blocks, followed by an initial structural optimization. Parameters for the warheads and building blocks were obtained separately using the antechamber module. While adding the linker to the ligand structure, the coordinates of the CRBN and BPTF ligands remained fixed, while the linker assumed an arbitrary conformation. This was supported by HeFei MiQro Era Digital technology Co., Ltd.

### Molecular dynamics simulation

Molecular dynamics simulations were conducted using the Amber22 software package, and the following force field models were applied: Amber ff14SB for the protein, TIP3P water model for water molecules, and the general amber force field 2 for small molecules. Each simulation box contained an average of approximately 25,500 water molecules, resulting in a total of 85,500 atoms. The structural optimization was initiated with a swift minimization comprising 13,000 steps. Subsequently, a gradual heating process to attain 300 K was executed in two phases. First, the system was heated from 0 to 100 K via a 50 ps simulation within the NVT ensemble. Subsequently, further heating occurred from 100 K to 300 K through a 200 ps simulation within the NPT ensemble. After the heating phase, equilibrium simulations were performed at 300 K for 1.25 ns with a time step of 2 fs, using the NPT ensemble. Finally, a 200 ns NVT production run, maintaining the temperature at 300 K, was carried out with a 2 fs time step. The optimal ternary complex conformation was determined by selecting the most prominent cluster from the clustering analysis of the equilibrium trajectory’s final 100 ns, using the Gromos clustering method.^54^ This was supported by HeFei MiQro Era Digital technology Co., Ltd.

### Molecular mechanics/generalized born surface area calculation method

To scrutinize the binding characteristics of the trajectories, the molecular mechanics/generalized born surface area (MM/GBSA) method was used. This methodology has been widely used in the analysis of PROTAC systems. The MM/GBSA binding free energy (ΔG_TEP_) was computed as follows (Eq. 1):

(Eq. 1) $$\Delta G_{TE_{,}P}=\Delta G_{TEP}-\Delta G_{TE}-G_{P}$$

In this equation, *T*, *P*, and *E* symbolize the target protein, PROTAC molecule, and E3 ligase, respectively. The *TE*, *P* pathway considers that the binary complex (TE) recruits the PROTAC (P) to form the ternary complex (TEP). Snapshots were extracted from each trajectory every 5 ns, and MM/GBSA scoring of ternary binding energies was performed using the MMPBSA.py module within Amber22. Implicit solvation was estimated using the generalized Born model,^55^ while the nonpolar solvation energy was calculated using the solvent-accessible surface area algorithm. It is worth noting that entropy contributions were omitted in the ΔG calculation to minimize potential sources of additional error.^56^ This was supported by HeFei MiQro Era Digital technology Co., Ltd.

### Cell lines and cell culture

Huh7 cells, verified by STR profiling test, were obtained from the Cell Bank/Stem cell Bank of the Chinese Academy of Sciences (Shanghai, China). Huh7 cells were cultured in DMEM (HyClone) supplemented with 10% heat-inactivated fetal bovine serum (FBS; Gibco) and 1% penicillin/streptomycin (Gibco). Human primary NK cells were cultured in complete RPMI 1640 medium containing IL-2 (100 U mL^−1^) and IL-15 (10 ng mL^−1^). The cells were maintained in humidified incubator at 37°C and 5% CO_2_.

### Dissociation and culture of primary HCC cells isolated from tumor tissues

Primary HCC cells were dissociated with the Tumor Dissociation Kit (Miltenyi Biotec, Cat: 130-095-929) according to the manufacturer’s instructions. After dissociation, the cells were cultured in DMEM supplemented with 10% FBS (Gibco), 1% penicillin/streptomycin (Gibco), and ITS-G (Pricella, PB180429).

### Total cell lysates extraction

Cells were harvested, washed twice with cold PBS, and centrifuged at 500×*g* for 5 min at 4°C. Subsequently, the cell pellets were lysed in the lysis buffer (25 mM Tris, pH 7.4, 150 mM NaCl, 10% glycerol, and 1% Nonidet P-40) supplemented with a protease inhibitor cocktail (Roche). After centrifugation at 14,000 × *g* for 20 min at 4°C, the supernatant was collected and used for western blot.

### Western blot

Protein samples were separated by 10% SDS-PAGE and then transferred onto a PVDF membrane. The membrane was blocked with 5% non-fat milk in TBST buffer (25 mM Tris, pH 7.4, 150 mM NaCl, 0.1% Tween 20) for 1 h at room temperature. The membrane was incubated with primary antibodies, which were diluted in TBST containing 5% BSA overnight at 4°C. After being washed three times with TBST, the membrane was incubated with horseradish peroxidase (HRP)-conjugated secondary antibodies in TBST containing 5% BSA for 1 h at room temperature. Subsequently, the membrane was washed three times with TBST and visualized using western blotting detection reagents (Bio-Rad, #1705061) on an Azure 300 imager (Azure Biosystems). The images were analyzed using ImageJ software. The primary antibodies used were BRD9 Rabbit mAb (CST, #58906), CECR2 Mouse mAb (Santa Cruz Biotechnology, sc-514878), BPTF Rabbit mAb (Abcam, ab72036), HPSE Mouse mAb (Santa Cruz Biotechnology, sc-515935), HS Mouse mAb (Millipore, #MAB2040), CRBN Polyclonal antibody (Proteintech, 28494-1-AP), anti-ubiquitin mouse mAb (NT) (PTM BIO, #PTM-5798), β-actin (8H10D10) Mouse mAb (Cell Signaling Technology, #3700). Secondary antibodies used were anti-rabbit IgG HRP-linked antibody (#7074) and anti-mouse IgG HRP-linked antibody (#7076) from Cell Signaling Technology.

### Calculation of DC_50_

The Huh7 Cells were treated with increasing concentrations of **8d** for 24 h. Subsequently, the expression levels of BPTF and β-actin were determined using western blot analysis and quantified using ImageJ software. The BPTF expression was normalized against the expression level of β-actin. The baseline BPTF expression in cells treated with DMSO was set as 100%. The relative BPTF expression level in cells treated with **8d** was calculated as a percentage relative to that in cells treated with DMSO.

### Isolation of primary NK cells

Primary NK cells were isolated from peripheral blood mononuclear cells (PBMCs). Blood samples were diluted 1:1 with PBS, then carefully layered over an equal volume of Ficoll in 15-mL conical tubes. The tubes were centrifuged at 1,000×*g* for 30 min at room temperature. The PBMC layer was carefully harvested and transferred to a new tube. Subsequently, the NK Cell Isolation Kit (Miltenyi Biotec, Cat. No. 130-092-657) was used according to the manufacturer’s protocol to purify primary NK cells.

### NK cell-mediated cytotoxicity assays

Target cells, including Huh7 cells or primary HCC cells, were pre-treated with DMSO (control), 16, 17, TP238, or **8d** for 24 h. Following this, these target cells were co-cultured with primary NK cells (effector cells) at an effector-to-target ratio of 1:20 in complete RPMI 1640 medium within 96-well plates for 4 h. Apoptosis of the target cells was assessed using Annexin V and 7-AAD double-staining following the manufacturer’s instructions (BD Biosciences, Cat. Nos. 556419 and 559925).

### Flow cytometry

Huh7 cells or primary HCC cells were pretreated with 10 μM of **8d**, 10 μM of TP238, or DMSO for 24 h. Subsequently, co-culture with human primary NK cells was carried out at the indicated E: T ratio for 4 h. Huh7 cells were stained with Annexin V (BD Biosciences, #556419) and 7-AAD (BD Biosciences, #559925). Human primary NK cells were stained with CD56-FITC (BD Biosciences, #557699), CD56-PE-Cy7(BD Biosciences, #557747), CD56-BV421(Biolegend, #362551), CD69-APC (BD Biosciences, #560711), CD69-FITC (BD Biosciences, #555530), NKG2D-PE-Cy7 (BD Biosciences, #562365), NKp30-APC (BD Biosciences, #558408), NKp44-PE-Cy7 (Biolegend, #325116), NKp46-PE (BD Biosciences, #557991), IFN-γ-FITC (Biolegend, #506504), TNF-α-PE (559321), GZMB-FITC (BD Biosciences, #560211), Perforin-PE (BD Biosciences, #556437), Perforin-BV510 (Biolegend, #308120), CD107a-APC (BD Biosciences, #560664), Granzyme B-BV421 (BD Biosciences, #563389). Mouse primary NK cells were stained with CD45-APC-Cy7 (Biolegend, #103116), CD3-PE-Cy7 (Biolegend, #100320), NKP46-PE-Cy7 (Biolegend, #137618), IFN-PerCP-Cy5.5 (Biolegend, #505822), TNF-α-BV421 (Biolegend, #506328), GZMB-PE (Biolegend, #372208), Perforin-APC (eBioscience, #17-9392-80), NKG2D-APC (eBioscience, #17–5882), CD107a-FITC (BD, #553793), CD3-PE (BD, #553064), or NK1.1-BV510 (BD, #563096) according to the manufacturer’s instructions. For intracellular staining, cells were initially incubated with antibodies against cell-surface markers at room temperature for 30 min. After adding 1 mL of PBS, the samples were centrifuged at 500 × *g* for 4 min at 4°C to remove any precipitates. Subsequently, the cells were fixed and permeabilized using a fixation/permeabilization buffer (Thermo Fisher Scientific, Cat: 00-5523-00) at room temperature for 30 min. Finally, the cells were stained with GZMB-BV421, perforin-BV510, IFN-PerCP-Cy5.5 and TNF-α-BV421, in darkness for an additional 30 min. The stained cells were then detected using a BD Fortessa flow cytometer (Figure S18) and analyzed with FlowJo software.

### Generation of BPTF KD Huh7 cells

Human BPTF siRNA (Horizon Discovery) was transfected into Huh7 cells using Lipofectamine RNAiMAX transfection reagent (Thermo Fisher Scientific, Cat: 13778075) according to the manufacturer’s instructions. Then, the cells were incubated in a humidified 37°C incubator with 5% CO_2_ for 48 h.

### High-performance liquid chromatography-tandem mass spectrometry analysis

Samples were analyzed on Easy-nLC 1200 liquid chromatography system (Thermo Fisher Scientific) coupled to Orbitrap Exploris 480 via a nano-electrospray ion source (Thermo Fisher Scientific). Dried peptide samples were dissolved in solvent A (0.1% formic acid in water) and loaded onto a trap column (100 μm × 2 cm, home-made; particle size, 3 μm; pore size, 120 Å; SunChrom) with a maximum pressure of 280 bar using solvent A, then separated on home-made 150 μm × 12 cm silica microcolumn (particle size, 1.9 μm; pore size, 120 Å; SunChrom) with a gradient of 5%–35% mobile phase B (acetonitrile and 0.1% formic acid) at a flow rate of 600 nL/min for 150 min. The eluted peptides were ionized and detected using high-field asymmetric waveform ion mobility spectrometry coupled with OE 480 MS (Thermo Fisher Scientific). MS analysis was conducted with one full scan (300–1,400 m/z, R = 120,000 at 200 m/z) at an automatic gain control target of 3e6 ions, followed by up to 20 data-dependent tandem mass spectrometry (MS/MS) scans with higher-energy collision dissociation (target 5e4 ions, max injection time 20 ms, isolation window 1.6 m/z, normalized collision energy of 27%). Data were acquired using the Xcalibur software (Thermo Fischer Scientific).

### MS quantification of proteins

MS raw files generated by liquid chromatography MS/MS were searched against the human National Center for Biotechnology Information RefSeq protein database using MaxQuant software. The protein abundance was estimated with a traditional label-free, intensity-based absolute quantification algorithm.^57^^,^^58^

### SPR-based binding assays

Biacore T200 instrument (GE Healthcare) was used to conduct the SPR binding assays and performed at 25°C. BPTF/BRD9/CECR2 bromodomain protein was immobilized on a NiHC1500M chip (XanTec bioanalytics, SCBS NiHC 1500M) after activated by injecting a solution of Ni^2+^ ions. First, the chip was equilibrated with PBS buffer, pH 7.4. For kinetic measurement, **8d** was diluted in PBS buffer at concentrations ranging from 0.3125 to 10 μM. **8d** was injected to bind with the protein for 60 s and dissociated for another 120 s. The KD value of **8d** against BPTF/BRD9/CECR2 bromodomain was determined by Biacore T200 evaluation software (GE Healthcare).

### RNA-seq and data analysis

To identify the genes regulated by BPTF in Huh7 cells, the cells were treated with BPTF siRNA or control siRNA for 72 h. Subsequently, the total RNA was extracted from the treated and control cells for RNA-seq. RNA-seq was performed and analyzed by BGI Shenzhen (two biological replicates per group). Raw read counts were used for differential gene expression analysis by DESeq2. Genes with an adjusted *p* value of less than 0.05 and log2FC R > 1.0 were considered as differentially expressed genes.

### RNA isolation and RT-qPCR

Total RNA was extracted from Huh7 cells using FastPure Cell/Tissue Total RNA Isolation Kit V2 (Nanjing Vazyme Biotech Co., Ltd, Cat: RC112-01), according to the manufacturer’s instructions. The RNA was reverse transcribed into cDNA using the HisScript III 1st Strand Synthesis Kit (+ gDNA wiper) (Nanjing Vazyme Biotech Co., Ltd, Cat: R312-01). qPCR was performed using the Taq Pro Universal SYBR qPCR Master Mix (Nanjing Vazyme Biotech Co., Ltd, Cat: R712-02) on an StepOnePlus Real-Time PCR System (Thermo Fisher Scientific, Inc.). The primer sequences used for qPCR are listed in Table S1. GAPDH was used as an internal reference gene. Relative FCs in mRNA expression were calculated using the formula 2^−ΔΔCt^.

### CUT&Tag-qPCR

DNAs were extracted from Huh7 cells and subjected to qPCR using Hyperactive Universal CUT&TAG Assay Kit for qPCR (Nanjing Vazyme Biotech Co., Ltd, Cat: TD904-01), according to the manufacturer’s instructions. The primer sequences used for CUT&Tag-qPCR were listed in Table S1.

### CUT&Taq for Illumina

Hyperactive Universal CUT&Tag Assay Kit for Illumina Pro (Nanjing Vazyme Biotech Co., Ltd, Cat: TD904-01) was used according to the manufacturer’s instructions. DNA sequencing performed by the Novogene Bioinformatics Technology Co., Ltd. Clean reads were aligned to the human reference genomes GRCh38 through bowtie2 software, and then the SAM files were converted into BAM files through bedtools. The BAM file is further converted into a bigwag file through deeptools, and the bigwig file is imported into the IGV software for visualization to obtain the target results.

### IP assay

Proteins were incubated with a specific rabbit polyclonal antibody against BPTF (Abcam, ab72036) or a nonspecific rabbit IgG (abcam, ab207-01) in IP buffer with gently rotation at 4°C for 4 h. Subsequently, Protein A/G agarose beads (Santa Cruz Biotechnology, Inc., sc-2003) were added to capture the antibody-protein complexes, and the incubation continued overnight at 4°C with rotation. The beads were then washed with washing buffer (25 mM Tris, pH 7.4, 150 mM NaCl, 0.2% Nonidet P-40) and subsequently resuspended in SDS-PAGE loading buffer to prepare samples. The samples were heated to 95°C for 10 min, followed by centrifugation to collect the supernatant for electrophoresis.

### Human samples

All human samples used in this study were obtained under the approval of the Ethics Committee of University of Science and Technology of China (USTCEC201600004; Hefei, China), and written informed consent was obtained from all patients. Table S2 provides clinical characteristics of these patients.

### Mice

Male C57BL/6 WT mice and female NCG mice (8 weeks old) were purchased from GemPharmatech and the Shanghai SLAC Laboratory Animal Co., Ltd. All mice were housed under specific pathogen-free conditions. All experimental procedures involving mice were conducted in accordance with the National Guidelines for Animal Usage in Research (China) and received approval from the Ethics Committee of University of Science and Technology of China (reference: USTCACUC26060124052).

### Establishment of the HCC model and **8d** treatment

The HCC cells used in this study were obtained from primary mouse HCC models generated through hydrodynamic tail-vein delivery of sgPten + Kras^G12D^ as previously described.^50^ These cells were generously provided by Prof. Haiming Wei from the University of Science and Technology of China. The process of orthotopic transplantation involved using C57BL/6 male mice and directly injecting 500,000 murine HCC cells into the subcapsular area of their left lateral liver lobe parenchyma. To prevent tumor cell leakage and subsequent peritoneal cavity metastasis, we suspended the cells in PBS before injection. The cell suspension was slowly injected to minimize damage to surrounding liver tissue and avoid leakage. Following the injection, Gelfoam was applied to cover the needle tract site to reduce bleeding and potential backflow or leakage. For **8d** treatment, intraperitoneal injection of either 10 mg/kg of **8d** in 0.2 mL corn oil (**8d** group) or the same volume of corn oil alone (control group) daily for 20 days. Finally, all of the mice were sacrificed at the specified time points.

### NK cell depletion mice model

For NK cell depletion experiments, mice were intraperitoneally injected with 300 μg of mouse anti-NK1.1 antibody (Bioxcell, BE0036) or 300 μg of mouse IgG2a isotype control (Bioxcell, cat. BE0085, Lot 833922A2) every 3 days.

### Human hepatoma model and NK cell transfer

The human hepatoma model was established by injection (intra-peritoneal) of human Huh7-luciferase cells (5 × 10^6^) into female NOD/ShiLtJGpt-Prkdc^em26Cd52^IL-2rg^em26Cd22^/Gpt (NCG, procured from GemPharmatech). After 2 days, we transferred 2 × 10^6^ human primary NK cells. To support NK cell survival *in vivo*, IL-2 (50,000 U) was intraperitoneally injected every 2 days. For tumor imaging *in vivo*, d-luciferin was injected (intraperitoneally) and imaged using an IVIS Spectrum imaging system (PerkinElmer).

### B-mode ultrasound imaging

A Vinno 6 performance real-time ultrasound scanner (Vinno Corporation) was used for ultrasound measurements. The tumor-bearing mice were anesthetized via intraperitoneal injection of 2% pentobarbital sodium and positioned on a warm platform to maintain euthermia. The abdominal hair was removed with a depilatory agent. Pre-warmed ultrasound gel was applied to the depilated abdomen skin. B-mode ultrasound imaging was used to display the maximum cross-sectional areas on day 13 and day 20 after tumor implantation in a 2D grayscale image.

### Histology and immunohistochemistry analysis

All tissues were initially fixed in 4% neutral-buffered formalin overnight for histology and immunohistochemical staining. Subsequently, 6-μm-thick sections were carefully cut and stained with hematoxylin and eosin. For immunohistochemistry analysis, liver tissues were deparaffinized and heat-induced antigen unmasking was conducted. Immunohistochemical stainings were carried out with the following antibodies: BPTF Rabbit mAb (Abcam, #ab72036); HPSE (E−10) Mouse mAb (Santa Cruz, #sc-515935).

### Statistical analysis

The statistical analysis was carried out using GraphPad Prism 7.0 software to assess the differences between experimental groups. The significance of the results was determined by a two-tailed Student’s t test and expressed as a *p* value. The statistical significance of group differences was determined according to the following criteria: ∗*p* < 0.05, ∗∗*p* < 0.01, ∗∗∗*p* < 0.001 and ∗∗∗∗*p* < 0.0001, respectively. When there was no significant difference between groups, it was indicated as *ns*.

## Data availability

Proteome raw have been deposited to the iProX partner repository (https://www.iprox.cn/) under Project ID: IPX0006605001. The link access to the raw data as follows: https://www.iprox.cn/page/SSV024.html;url=1697962492604W6nd.

## Acknowledgments

This project was supported by the 10.13039/501100001809National Natural Science Foundation of China (No. 22277115) and Strategic Priority Research Program of the Chinese Academy of Sciences (XDB0940202) (Y.W.); The Anhui Natural Science Foundation (2408085MH193) (J.J.); 10.13039/501100012166National Key R&D Program of China (2017YFA0505102) and 10.13039/501100001809National Natural Science Foundation of China (31770886, 31972933) (C.D.); HK-RGC grant GRF17104120 (Q.H.); and The Global Select Project of the Institute of Health and Medicine, Hefei Comprehensive National Science Center (DJK-LX2022003) (Y.W.).

## Author contributions

Y.W., J.J., and C.D. conceived the idea. Y.L., L.B., H.L., P.Y., and ZH.C. performed all the experiments and analyzed the data. H.C. provided the clinical human samples. All authors contributed to drafting and revising the manuscript.

## Declaration of interests

The authors declare that they have no competing interests.

## Supplemental information

Document S1. Figures S1–S18, Tables S1, and S2

Document S2. Article plus supplemental information
