# Supplementary material for: A BPTF-specific PROTAC degrader enhances NK cell-based cancer immunotherapy
Source: Mol Ther. 2025 Feb 11;33(4):1566–83. doi: 10.1016/j.ymthe.2025.02.013 (PMC11997503; doi:10.1016/j.ymthe.2025.02.013)
Supplement: Document S1. Figures S1–S18, Tables S1, and S2 [file mmc1.pdf]

YMTHE, Volume *33*

Supplemental Information

A BPTF-specific PROTAC degrader enhances NK cell-based cancer immunotherapy

Yunjia Li, Lin Bai, Hao Liang, Peidong Yan, Hao Chen, Zhuoxian Cao, Yiqing Shen, Zhongyv Wang, Mei Huang, Bin He, Quan Hao, Yide Mei, Haiming Wei, Chen Ding, Jing Jin, and Yi Wang

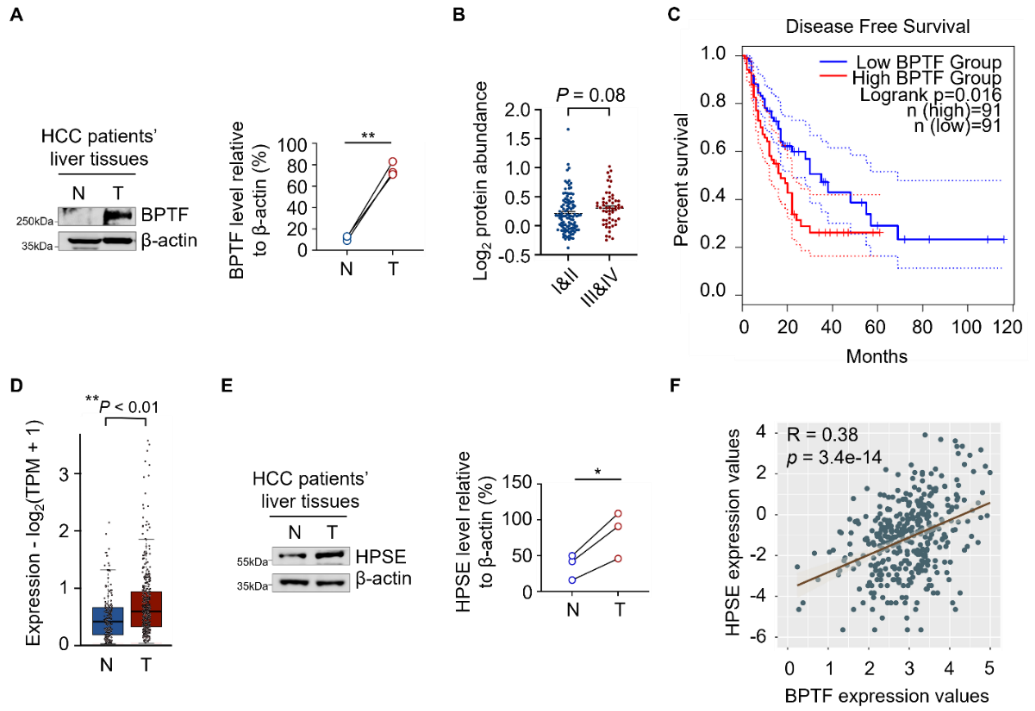

Figure S1. The expressions and associations of BPTF and HPSE in the tumor tissues of HCC patients.

A. Immunoblot analysis (left) and quantification (right, n = 3) of BPTF in the lysates isolated from HCC patients' tumor (T) and adjacent non-tumor tissues (N). β-actin was used as the loading control.

B. Comparisons of the protein abundance of BPTF between I & II stage and III & IV stage of tumor tissue samples (unpaired *t*-test) in the HCC patients' cohort (n = 159). The line represents mean with SEM and upper and lower quartiles, respectively. The *P*-value is depicted in the figure.

C. Disease-free survival analysis in a separate subset of 91 HCC patients with either low (blue) or high (red) BPTF expression sourced from the GEPIA2 database (http://gepia2.cancer-pku.cn/#survival).

D. Relative HPSE expression abundance in tumor tissue and adjacnet tissue in HCC patients's cohot (T = 369, N = 160) from TCGA database (https://www.cancer.gov/ccg/research/genome-sequencing/tcga).

E. Immunoblot analysis (left) and quantification (right, $n = 3$) of HPSE in the lysates isolated from HCC patients' tumor (T) and adjacent non-tumor tissues (N). β-actin was used as the loading control.

F. Scatterplot showing the relationship between the expression level of BPTF and HPSE in the tumor tissues of HCC patients.

The data in **A**, **E** were presented as mean ±SD; $^{*}P < 0.05$, $^{**}P < 0.01$, $^{***}P < 0.001$, $^{****}P < 0.0001$; Paired *t*-test.

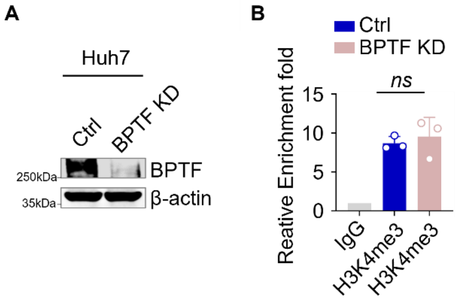

Figure S2. The analysis of CUT&Tag-qPCR in control and BPTF knockdown (KD) Huh7 cells.

A. Immunoblotting of BPTF expression in the total cell lysates isolated from Huh7 cells treated with or without BPTF siRNA. β-actin was used as a loading control.

B. CUT&Tag-qPCR analysis using anti-H3K4me3 antibody and IgG control to detect the enrichment of H3K4me3 at the promoter region of *HPSE* in control and BPTF KD Huh7 cells (n = 3). The data was presented as mean ± SD; *ns* (no significance, $P \geq 0.05$); Unpaired t-test.

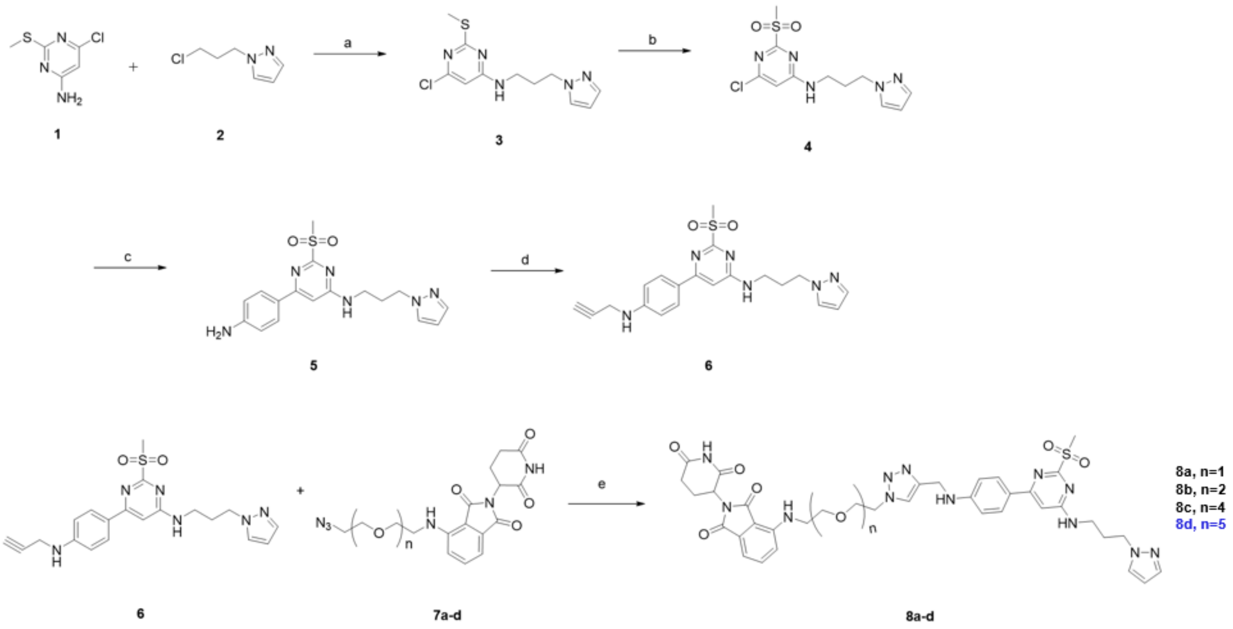

Figure S3. Synthesis of BPTF PROTACs 8a-d.

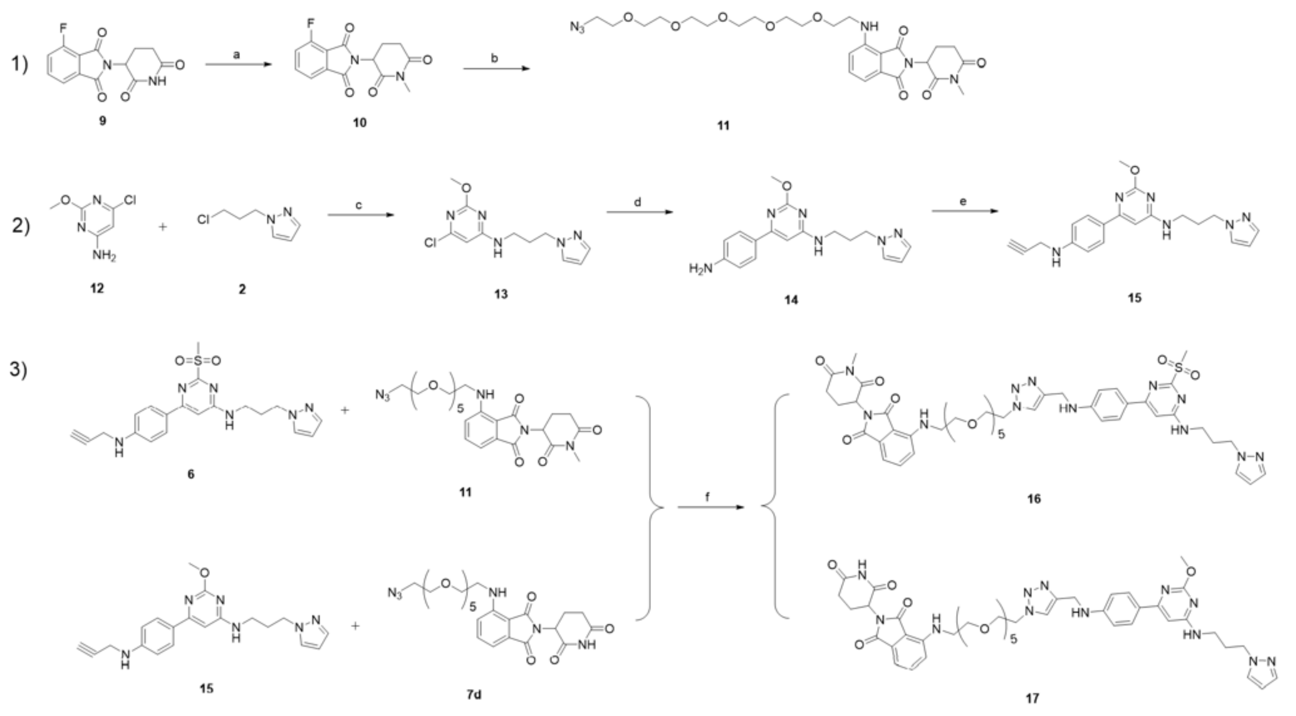

Figure S4. Synthesis of Deactivated Degraders 16-17.

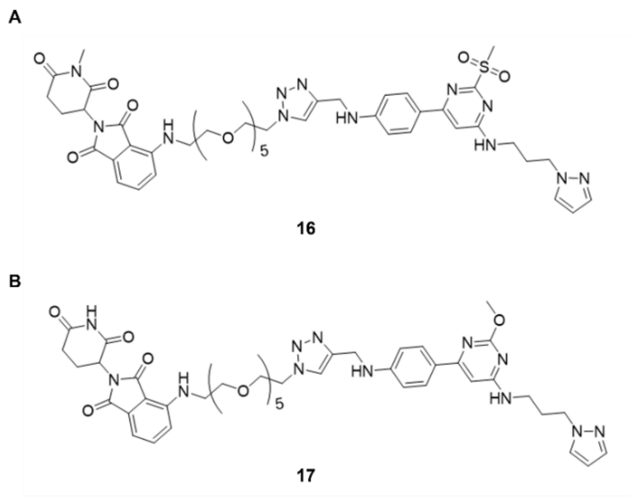

Figure S5. The structures of deactivated degraders 16 and 17

A

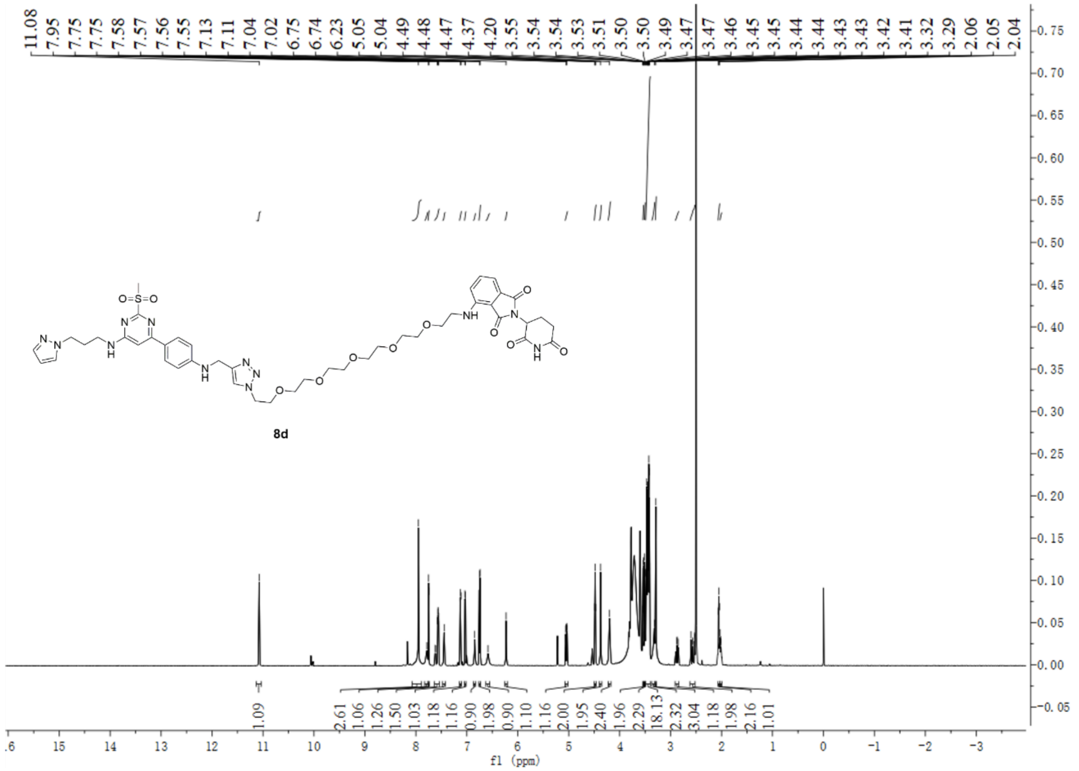

B

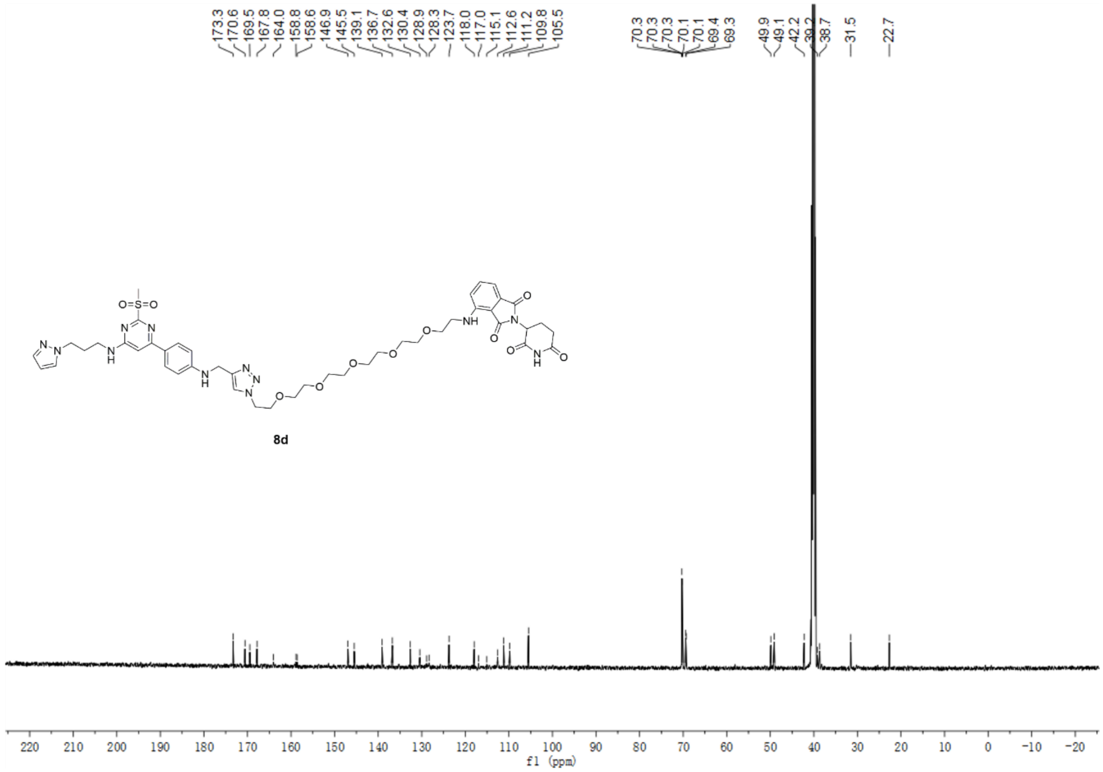

C

Chromatogram and Results

Injection Details

Injection Name:	**028-1**	*Run Time (min):*	**30.00**
Vial Number:	**4**	*Injection Volume:*	**100.00**
Injection Type:	**Unknown**	*Channel:*	**UV_VIS_4**
Calibration Level:		*Wavelength:*	**365**
Instrument Method:	**1mL-1**	*Bandwidth:*	**n.a.**
Processing Method:	**Basic Quantitative**	*Dilution Factor:*	**1.0000**
Injection Date/Time:	**18/Jun/22 16:15**	*Sample Weight:*	**1.0000**

Chromatogram

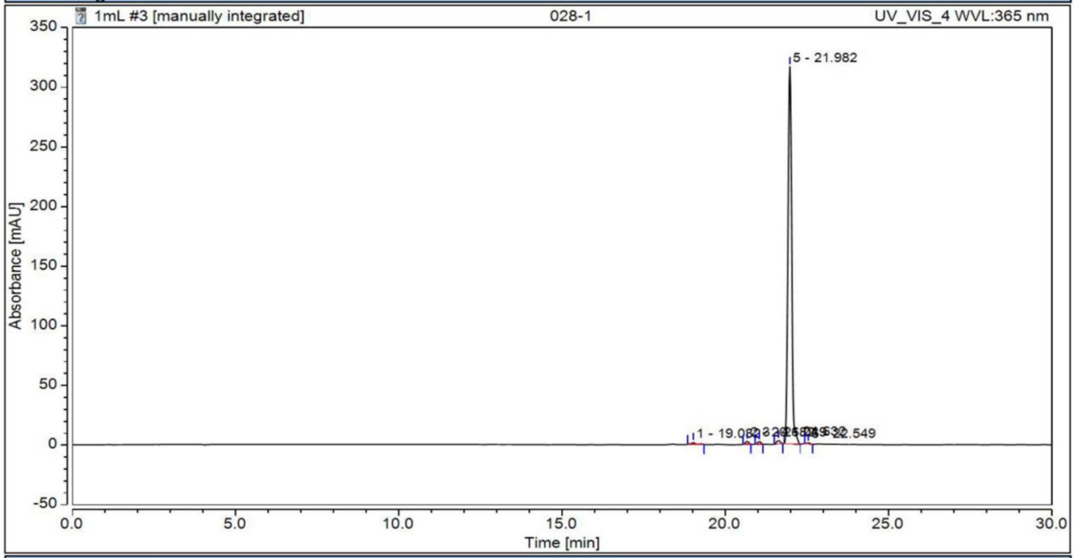

Integration Results

No.	Peak Name	Retention Time min	Area mAU*min	Height mAU	Relative Area %	Relative Height %	Amount n.a.
1		19.032	0.277	1.445	0.64	0.44	n.a.
2		20.682	0.291	2.598	0.67	0.80	n.a.
3		21.049	0.258	2.340	0.60	0.72	n.a.
4		21.632	0.382	3.054	0.89	0.94	n.a.
5		21.982	41.801	316.219	96.97	96.83	n.a.
6		22.549	0.098	0.918	0.23	0.28	n.a.
**Total:**			**43.106**	**326.573**	**100.00**	**100.00**	

D

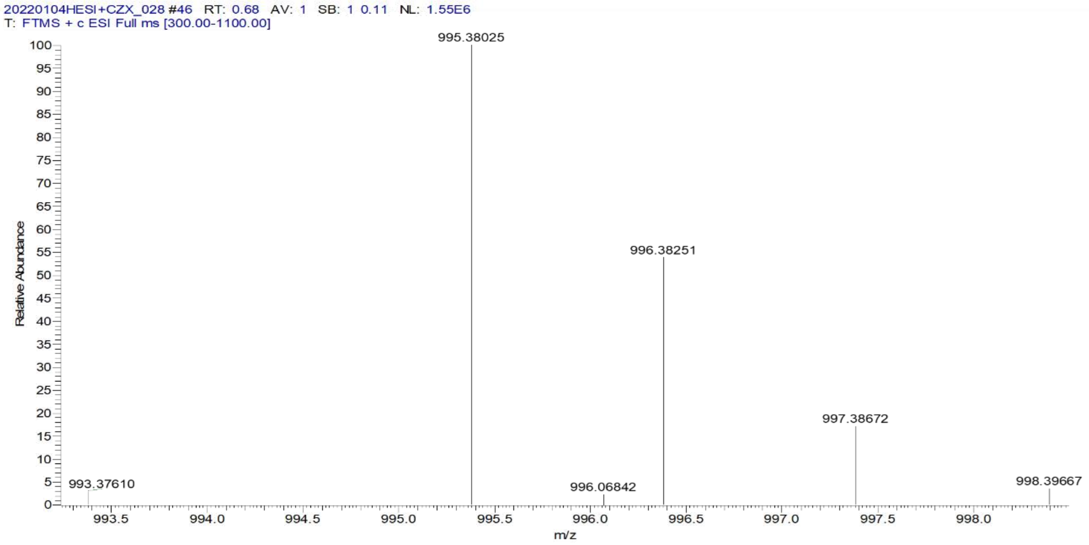

E

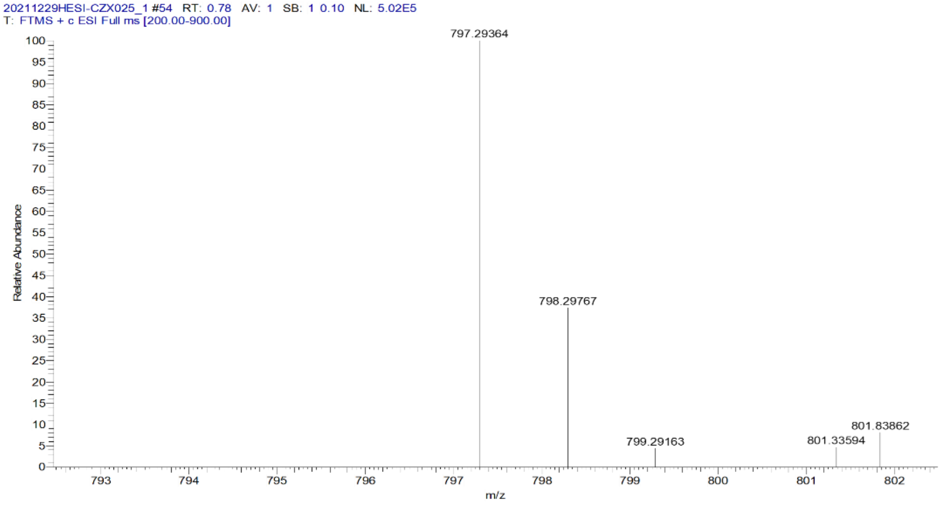
20211229HESI-CZX025_1 #54 RT: 0.78 AV: 1 SB: 1 0.10 NL: 5.02E5
T: FTMS + c ESI Full ms [200.00-900.00]
797.29364
798.29767
799.29163
801.33594
801.83862
Relative Abundance
m/z

F

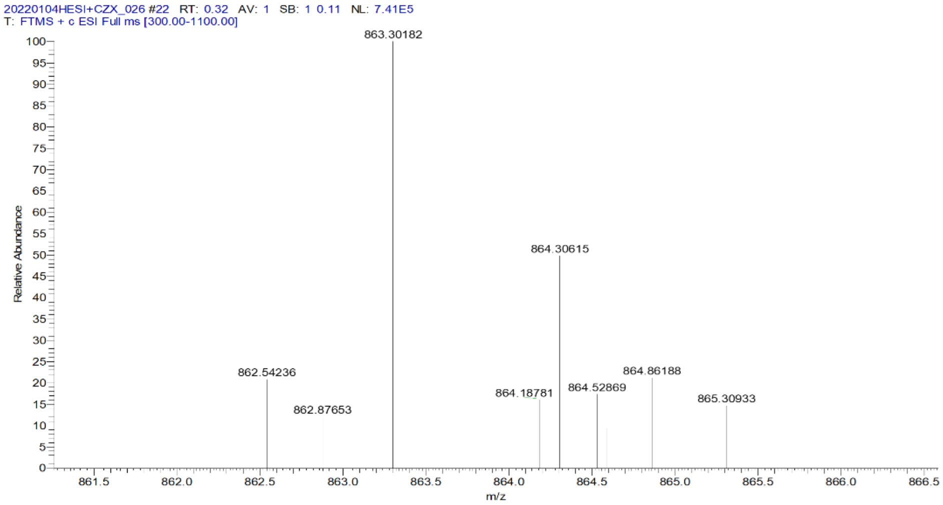
20220104HESI+CZX_026 #22 RT: 0.32 AV: 1 SB: 1 0.11 NL: 7.41E5
T: FTMS + c ESI Full ms [300.00-1100.00]
863.30182
864.30615
862.54236
862.87653
864.18781
864.52869
864.86188
865.30933
Relative Abundance
m/z

G

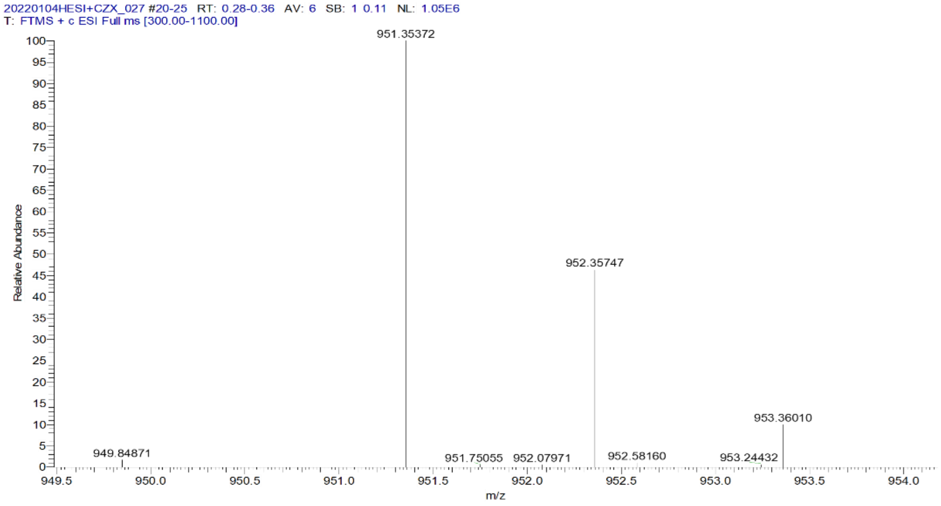
20220104HESI+CZX_027 #20-25 RT: 0.28-0.36 AV: 6 SB: 1 0.11 NL: 1.05E6
T: FTMS + c ESI Full ms [300.00-1100.00]
951.35372
952.35747
953.36010
949.84871
951.75055
952.07971
952.58160
953.24432
Relative Abundance
m/z

H

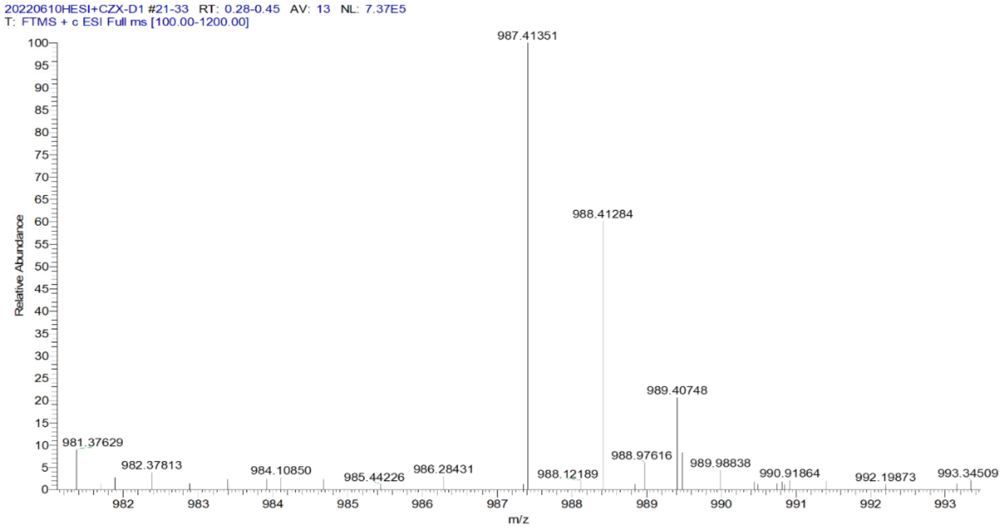

I

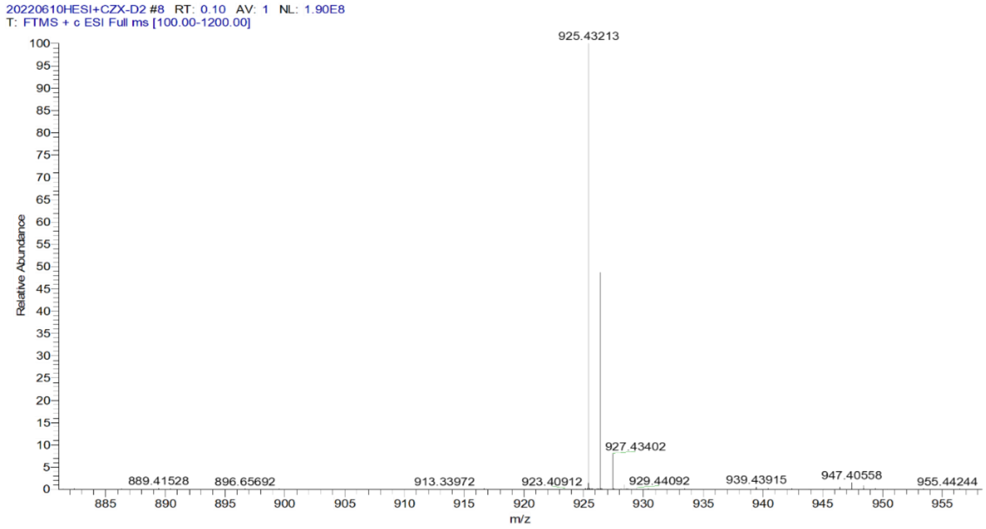

Figure S6. Identification of all compounds.

A. ^{1}H NMR Spectrum of **8d**.

B. ^{13}C NMR Spectrum of **8d**.

C. HPLC purity analysis for compound **8d**.

D-I. MS spectrum of **8d** (D); **8a** (E); **8b** (F); **8c** (G); **16** (H); **17** (I).

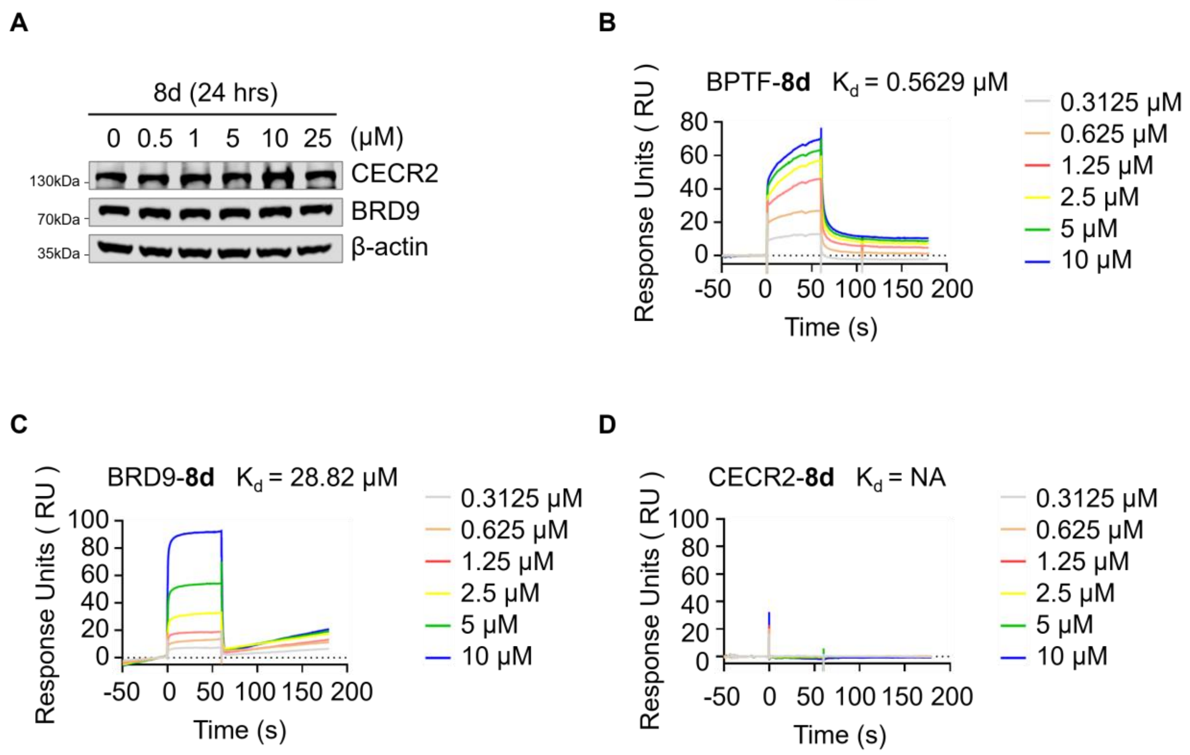

Figure S7. The binding affinities of BPTF, CECR2 and BRD9 bromodomains for 8d, measured by surface plasmon resonance (SPR) spectroscopy.

A. Immunoblotting of CECR2 and BRD9 in the total cell lysates. The lysates were isolated from Huh7 cells treated with **8d** at the indicated doses (0, 0.5, 1, 5, 10, 25 μM) for 24 hrs, respectively. β-actin was used as the loading control. The displayed image is representative of three biological replicates.

B-D. SPR traces for the binding interactions of **8d** with BPTF bromodomain (**B**), BRD9 bromodomain (**C**), and CECR2 bromodomain (**D**).

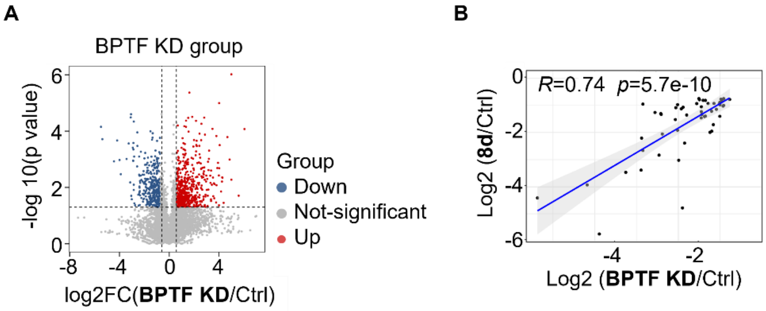

Figure S8. The proteomics analyses of 8d-treated Huh7 cells and BPTF KD Huh7 cells.

A. Volcanic plot showing the altered protein expression between control and BPTF KD Huh7 cells. Significantly downregulated proteins are depicted as blue dots and upregulated proteins as red dots (two-sided paired *t*-test, *P*-value < 0.05, FC > 1.5).

B. Correlation of downregulated proteins in **8d**-treated Huh7 cells and BPTF KD Huh7 cells.

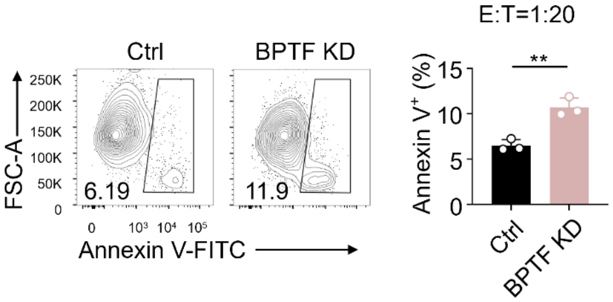

Figure S9. The cytotoxicity of NK cells towards BPTF KD Huh7 cells. Flow cytometry analysis (left) and quantification (right, $n = 3$) of the percentage of Annexin V^+ Huh7 cells (target cells) in a co-culture with human primary NK cells (effector cells) for 4 hrs, at an effector/target ratio of 1:20. The control Huh7 cells and BPTF-KD Huh7 cells were included in the experiment. The data was presented as mean ± SD; *$P < 0.05$, **$P < 0.01$, ***$P < 0.001$, ****$P < 0.0001$; Unpaired *t*-test.

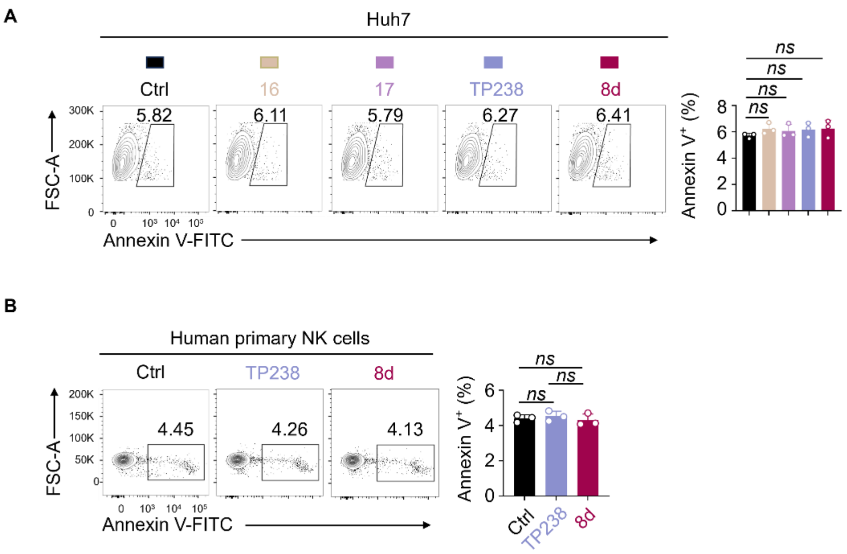

Figure S10. The effect of 8d toxicity on Huh7 or human primary NK cells.

A. Flow cytometry analysis showing the percentage of Annexin V^+ Huh7 cells that were treated with compound 16, 17, TP238 or **8d** at 10 μM for 28 hrs.

B. Flow cytometry analysis indicating the percentage of Annexin V^+ human primary NK cells that were treated with TP238 or **8d** at 10 μM for 4 hrs.

The data were presented as mean ± SD (*n* = 3); *ns* (no significance, $P \geq 0.05$); Unpaired *t*-test.

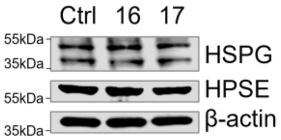

Figure S11. The effect of deactivated degraders on HSPG and HPSE expressions. Immunoblotting of HSPG and HPSE in the total cell lysates. The lysates were obtained from Huh7 cells treated with **16** (10 μM) or **17** (10 μM) for 24 hrs, and β-actin served as the loading control. The presented image is representative of three independent experiments.

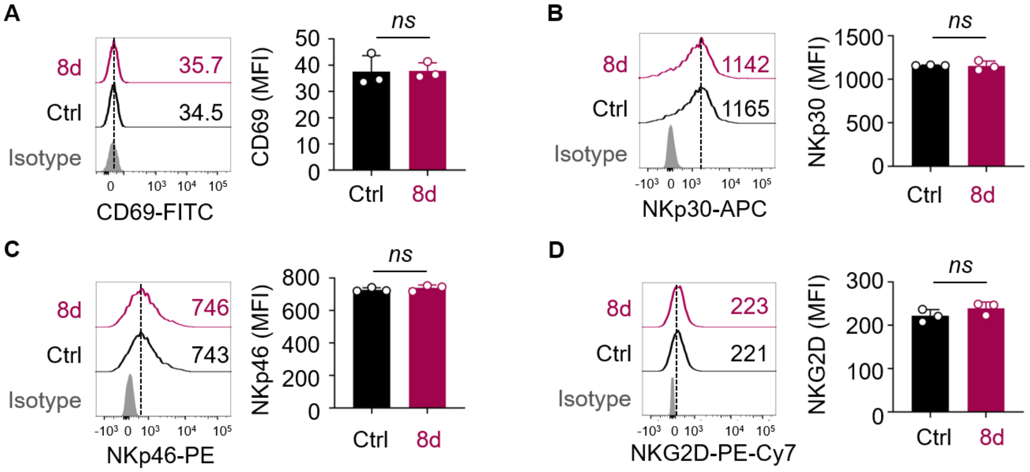

Figure S12. The effect of 8d on the activation of human primary NK cells.

A-D. Flow cytometry analysis showing the expression of CD69 (**A**), NKp30 (**B**), NKp46 (**C**), NKG2D (**D**) on human primary NK cells. The NK cells were treated with **8d** for 4 hrs. The data were presented as mean ± SD (n = 3); *ns* (no significance, $P \geq 0.05$); Unpaired t-test.

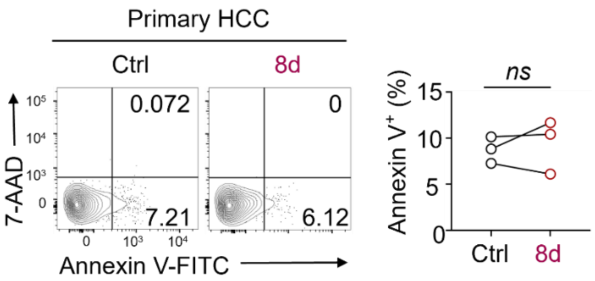

Figure S13. The effect of 8d toxicity on primary HCC cells isolated from the tumor tissues of HCC patients. Flow cytometry analysis indicating the percentage of Annexin V$^+$ primary HCC cells that were treated with or without **8d** at 10 μM for 28 hrs. The data were presented as mean ± SD (n = 3); *ns* (no significance, $P \geq 0.05$); Paired t-test.

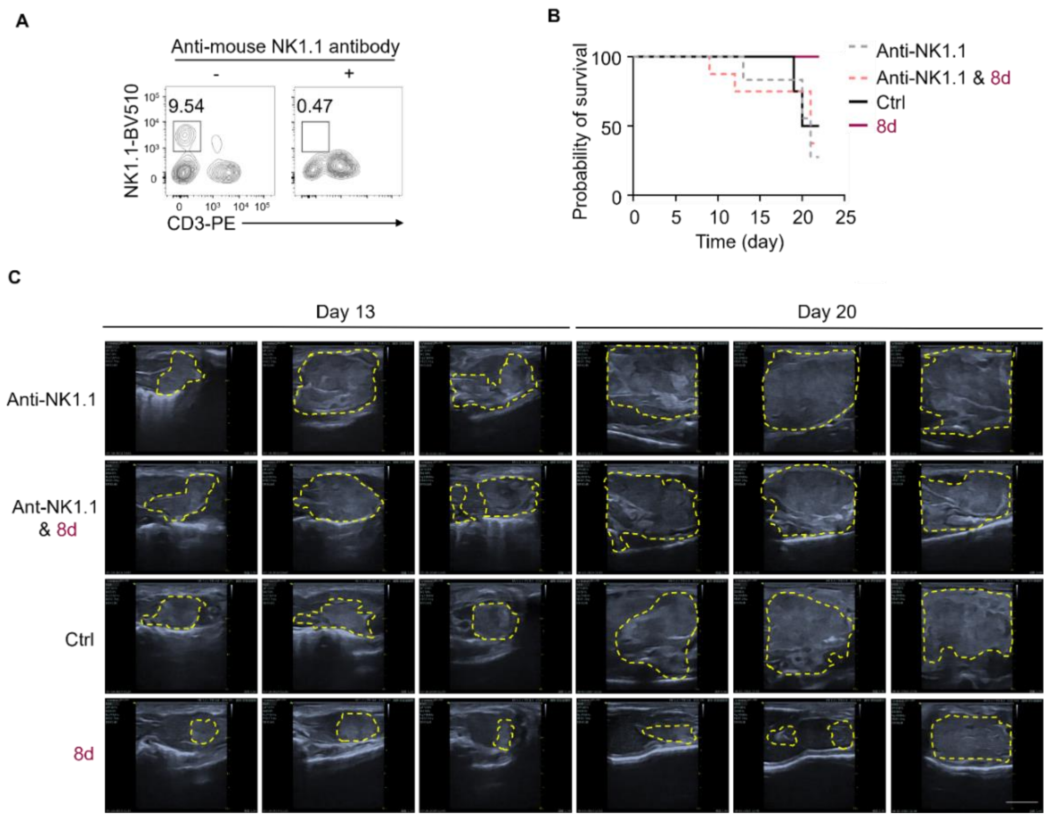

Figure S14. Treatment with 8d prolongs the survival and reduces the tumor size in HCC mice.

A. Flow cytometry analysis showing the percentage of NK cells from the liver tissue of HCC mice with or without NK depletion treatment.

B. Kaplan–Meier overall survival curves for four groups: HCC mice with NK cell depletion treated with vehicle group (grey), HCC mice with NK cell depletion treated with **8d** (pink), control HCC mice (black), and HCC mice treated with **8d** (purplish red).

C. B-mode ultrasound images of mouse livers at different progressive stages of HCC from four groups: HCC mice with NK cell depletion treated with vehicle (1st row), HCC mice with NK cell depletion treated with **8d** (2nd row), control HCC mice (3rd row), HCC mice treated with **8d** (4th row). $n = 3$ for each group. Yellow dotted areas indicate the HCC tumor border. Scale bars, 0.5 cm.

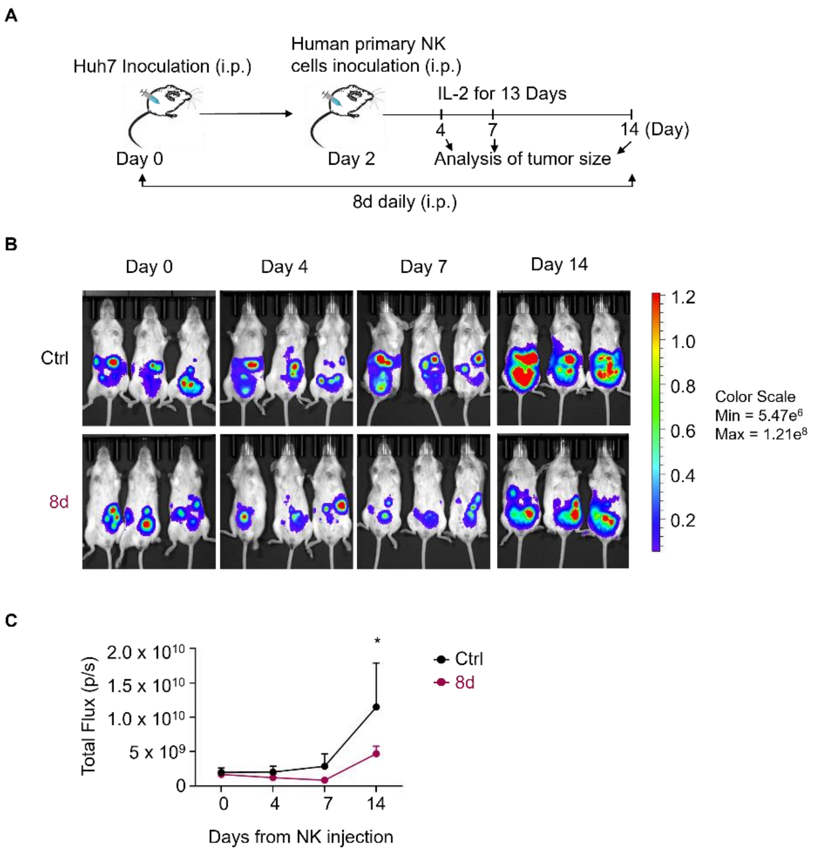

Figure S15. 8d treatment inhibits tumor growth in the human HCC mouse models.

A. Human HCC mouse models were established in NCG mice by intra-peritoneal injection of 5×10^6 human Huh7-luciferase cells, followed by the transfer of 2×10^6 human primary NK cells two days later. At the indicated day, for *in vivo* tumor imaging, D-luciferin was injected intra-peritoneally and imaged using an IVIS spectrum imaging system.

B. Bioluminescence imaging of Human HCC mouse models treated with or without **8d**. $n = 3$ for each group.

C. HCC burden was quantified as the average value of the total flux (p/s). $n = 3$ mice per group. The data were presented as mean ± SD (n = 3); $*P < 0.05$; Two-way ANOVA with multiple comparisons test.

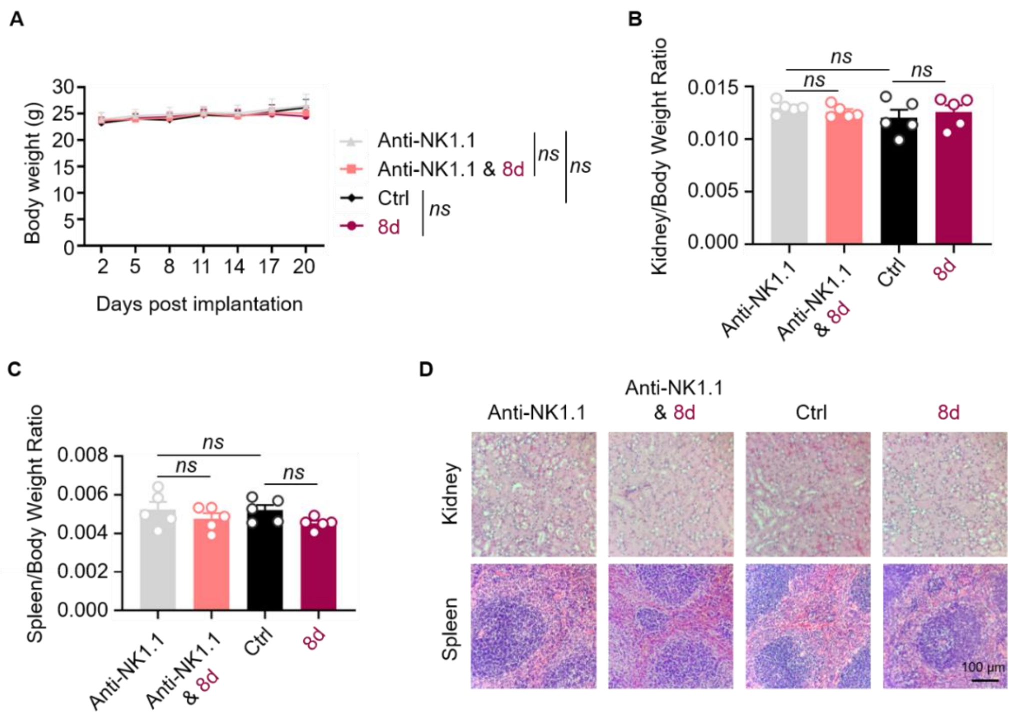

Figure S16. 8d treatment does not induce pathological damage in the kidney or spleen *in vivo*.

A. Body weights of individual mice in the indicated four groups from day 1 to day 20. The data were presented as mean ± SD (n = 5); *ns* (no significance, $P \geq 0.05$); Two-way ANOVA with multiple comparisons test.

B-C. Quantification of kidney/body weight (**B**) and spleen/body weight (**C**) ratios in mice from the four groups at autopsy on day 21. The data were presented as mean ± SD ($n = 5$); *ns* (no significance, $P \geq 0.05$); Unpaired *t*-test.

D. Representative hematoxylin and eosin (H&E) staining of kidney and spleen tissues harvested from the four groups. Scale bars, 100 μm. Data are representative of three independent experiments.

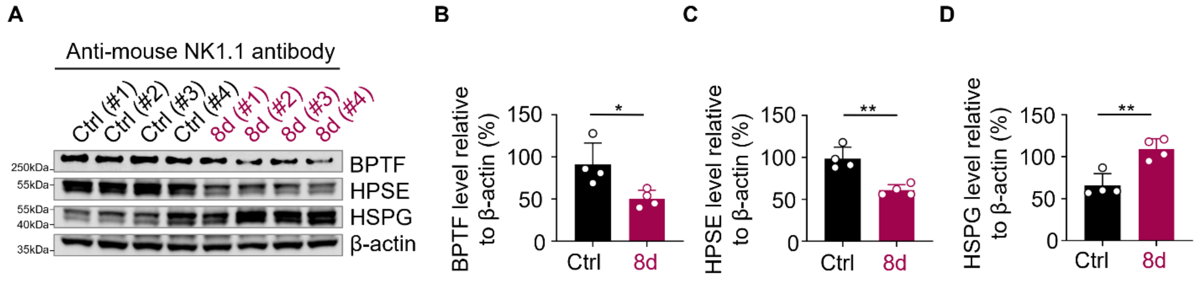

Figure S17. 8d-induced downregulation of BPTF in primary HCC cells of mice.

A-D. Immunoblot analysis and quantification (n = 4) of BPTF (**A, B**), HPSE (**A, C**), and HSPG (**A, D**) in the lysates extracted from the liver tissues of NK-depleted HCC mice treated with or without **8d**. β-actin was used as the loading control. The data were presented as mean ± SD; *$P < 0.05$, **$P < 0.01$, ***$P < 0.001$, ****$P < 0.0001$; Unpaired t-test.

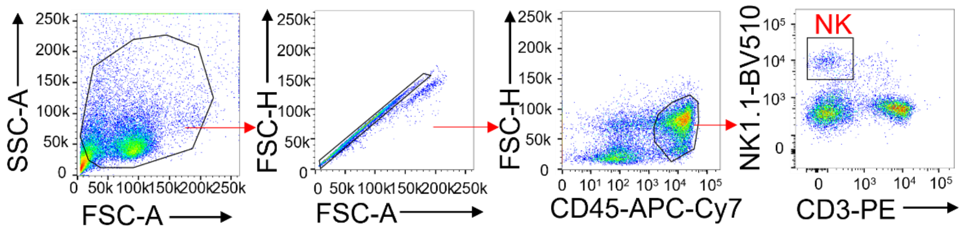

Figure S18. Gating strategy for primary NK cells in flow cytometry.

Table S1. Primer sequences

HPSE ChIP-qPCR_F: CCAGATGCCACCACAATGCAA
HPSE ChIP-qPCR_R: TACTCTCAGACCCCAAACTGCT
HPSE qPCR _F: TCATCCTCCTGGGTTCTCCA
HPSE qPCR _R: AGGGCCATTCCAACCGTAAC
GAPDH qPCR _F: CCATGGGGAAGGTGAAGGTC
GAPDH qPCR _R: GAAGGGGTCATTGATGGCAAC

Table S2. HCC Patients Characteristics

Patient	Gender	Age (years)	HBV	HCV	HBsAg^{+}	HCC	AFP (ng/mL)	ALT (u/L)	AST (u/L)
1	M	57	Y	N	Y	Y	3.82	26	30
2	M	60	Y	N	Y	Y	15.40	39	46
3	M	65	Y	N	Y	Y	192.12	43	66
4	M	75	Y	N	Y	Y	3.55	31	34
5	M	57	Y	N	Y	Y	10.73	42	28
6	M	54	Y	N	Y	Y	>2000.00	30	59
7	M	62	Y	N	Y	Y	1.96	27	25
8	M	54	Y	N	Y	Y	8.31	33	39
9	M	57	Y	N	Y	Y	3.32	36.2	31.2
10	M	54	Y	N	Y	Y	24.14	133	41
11	M	69	Y	N	Y	Y	>2000.00	24	23
12	F	70	Y	N	Y	Y	>2000.00	46	49
13	M	75	Y	N	Y	Y	10.20	44	34
14	M	72	Y	N	Y	Y	2	21	21
15	M	61	Y	N	Y	Y	127.97	26	21
16	M	31	Y	N	Y	Y	>2000.00	31	16

All patients were Chinese subjects. M, male; F, female； HBV, hepatitis B virus; HCV, hepatitis C virus; HBsAg, hepatitis B virus surface antigen; HCC, hepatocellular carcinoma; AFP, alpha-fetoprotein; ALT, Alanine aminotransferase; AST, Aspartate aminotransferase; N, no; Y, yes.
